# Ethylene modulates translation dynamics in *Arabidopsis* under submergence via GCN2 and EIN2

**DOI:** 10.1126/sciadv.abm7863

**Published:** 2022-06-03

**Authors:** Hsing-Yi Cho, Mei-Yi Chou, Hsiu-Yin Ho, Wan-Chieh Chen, Ming-Che Shih

**Affiliations:** Agricultural Biotechnology Research Center, Academia Sinica, Taipei 11529, Taiwan.

## Abstract

General translational repression is a key process that reduces energy consumption under hypoxia. Here, we show that plant stress-activated general control nonderepressible 2 (GCN2) was activated to regulate the reduction in polysome loading during submergence in *Arabidopsis*. GCN2 signaling was activated by ethylene under submergence. GCN2 activity was reduced in *etr1-1*, but not in *ein2-5* or *eil1ein3*, under submergence, suggesting that GCN2 activity is regulated by a noncanonical ethylene signaling pathway. Polysome loading was not reduced in *ein2-5* under submergence, implying that ethylene modulates translation via both EIN2 and GCN2. Transcriptomic analysis demonstrated that EIN2 and GCN2 regulate not only general translational repression but also translational enhancement of specific mRNAs under submergence. Together, these results demonstrate that during submergence, entrapped ethylene triggers GCN2 and EIN2 to regulate translation dynamics and ensure the translation of stress response proteins.

## Teaser

A noncanonical ethylene signaling pathway triggers GCN2 to modulate translation dynamics in *Arabidopsis* during submergence.

## INTRODUCTION

Translational reprograming under hypoxia has been observed for decades. In eukaryotic cells, once mRNAs are transported from nuclei to the cytosol, they may be translated, degraded, or stored (*1*–*4*). In plant cells under hypoxia, general translation is repressed, including cap-mediated translation (*5*, *6*). The untranslated mRNAs are transported into stress granules or processing (P) bodies (*7*–*9*). On the other hand, the mRNAs of a group of hypoxia response genes (HRGs) can bypass translational repression (*1*, *10*) and be translated under hypoxia; this process is controlled by a plant-specific SnRK1-eIFiso4G1 signaling cascade (*10*, *11*). However, it is still unclear how plants sense environmental signals to regulate the repression of general translation.

General control nonderepressible 2 (GCN2) is an evolutionarily conserved regulator that perceives nutrient starvation and stress signals (*12*, *13*). In yeasts and mammals, GCN2 senses amino acid starvation via its C-terminal region, histidyl tRNA synthetase, which stimulates the activity of the N-terminal kinase domain and turns on downstream signaling. GCN2 can phosphorylate eukaryotic initiation factor 2α (eIF2α) to stop the exchange of guanosine diphosphate (GDP) to guanosine triphosphate (GTP) by eIF2B and arrest general translation but initiate translation of specific mRNAs, such as *GCN4* in yeast (*14*) and *ATF* in mammals (*3*). *Arabidopsis* has one copy of *GCN2* and two copies of *eIF2*α. The phosphorylation site in eIF2α (Ser^56^) recognized by GCN2 is conserved and phosphorylated in response to diverse stimuli, including inhibitors of amino acid and purine biosynthesis, wounding, high light, ultraviolet (UV) light, hormones, and bacterial infection (*15*–*17*). However, there is still uncertainty about how similar the plant GCN2-eIF2α pathway is to that of yeast and mammals (*18*).

Ethylene is a key phytohormone involved in plant responses to hypoxic stress (*19*, *20*). In the early stage of submergence, ethylene is trapped in the submerged tissues due to restricted gas diffusion underwater (*21*). The rapid increase in ethylene triggers a signaling pathway that, through the elimination of nitric oxide, results in the stabilization of the group VII ethylene response factors (ERFs), leading to the activation of HRGs (*22*, *23*). Ethylene signaling induces the expression of genes that are involved in systemic signaling response and fermentation and triggers morphological and anatomical changes in some plant species (*24*, *25*). Under ethylene treatment, a C-terminal fragment of EIN2 suppresses the translation of *EBF1* and *EBF2* mRNAs, two EIN3-binding F box proteins, thereby limiting EIN3 and EIL1 turnover (*26*, *27*). Aminocyclopropane-1-carboxylic acid (ACC), the precursor of ethylene, enhances GCN2-mediated phosphorylation of eIF2α (*15*). These observations raise the possibility that the elevated ethylene levels under submergence might be involved in regulating GCN2/eIF2α signaling.

To investigate the functions of GCN2/eIF2α and ethylene in regulating mRNA loading onto ribosomes in *Arabidopsis*, we compared the transcriptomes and translatomes of ethylene mutants and *gcn2* under submergence. GCN2-mediated phosphorylation of eIF2α is enhanced under submergence, resulting in a reduction of polysome loading of mRNAs. This enhanced GCN2 activity is triggered through a noncanonical ethylene signaling pathway, in which the activity of GCN2 is independent of EIN2, EIN3, and EIL1. However, compared with the wild type, the general translational repression was less severe in either *ein2-5* or *gcn2* under submergence, indicating that ethylene triggers two pathways, one EIN2 dependent and the other GCN2 dependent, to regulate translation dynamics during submergence. Next-generation sequencing analyses indicated that, in addition to repressing translation, both GCN2 and EIN2 can regulate transcript levels and translation efficiency of specific mRNAs during submergence. Overall, our results showed that under submergence, the entrapped ethylene activates EIN2 and GCN2, causing a reduction of general translation and the production of stress response proteins, allowing plants to rapidly respond to low oxygen stress.

## RESULTS

### GCN2-eIF2α signaling mediates the repression of general translation during submergence

**Fig. 1.**
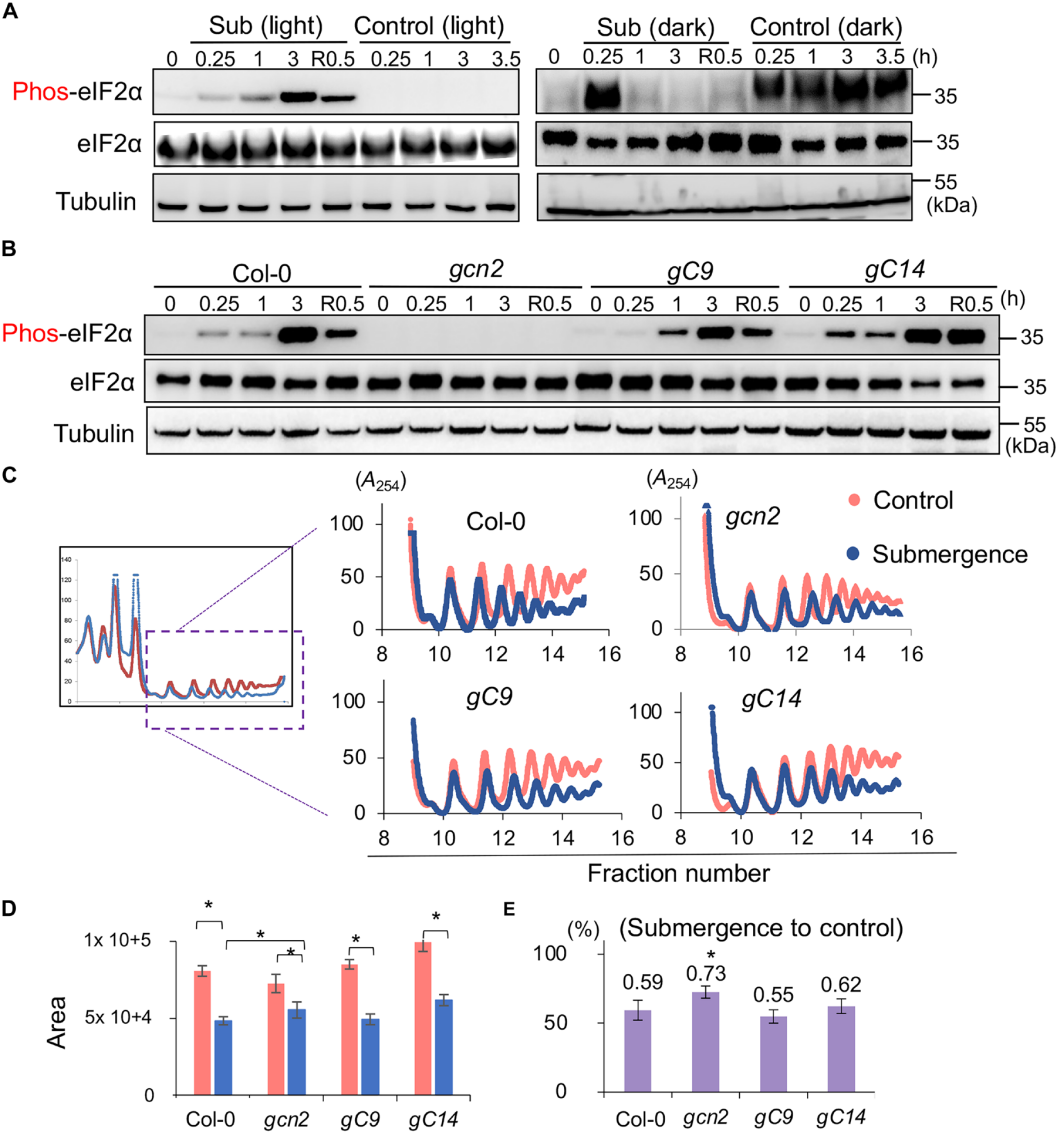
GCN2-eIF2α signal is triggered under submergence in *Arabidopsis*. (**A**) Western blots showing the phosphorylation profile of eIF2α in the whole seedlings of Col-0 under submergence (light) and control (light) (left panels), and submergence (dark) and control (dark) (right panels). Tubulin was used as the internal control. R, recovery. Three independent biological repeats showed a similar pattern. (**B**) Western blot showing phosphorylated eIF2α (panel one) and total eIF2α (panel two) in the whole seedlings of Col-0, *gcn2*, and two GCN2 overexpression lines in the *gcn2* background under submergence and recovery. Tubulin was used as the internal control. Three independent biological repeats showed a similar pattern. (**C**) The polysome profiles of Col-0, *gcn2*, and two GCN2 overexpression lines (*gcn2* background) under control (red line) and 1-hour submergence (light) (blue line) conditions. Three independent biological repeats showed a similar pattern. *A*_254_, absorbance at 254 nm. (**D** and **E**) Bar graphs showing (D) the area of polysome loading profile curves for the four lines in (C) under control and 1-hour submergence (light) conditions and (E) the ratio of submergence to control areas. The results represent the mean ± SEM of five biological replicates (two-tailed *t* test, *P < 0.05).

To examine whether GCN2-eIF2α signaling is activated in *Arabidopsis* under submergence, we examined the phosphorylation of eIF2α in long-day-grown Col-0 seedlings, using 2 hours after the beginning of the photoperiod, at which point no notable phosphorylation of eIF2α was detected (Fig. 1A, lane 1, time zero). Phosphorylation of eIF2α was induced in Col-0 under submergence (the final dissolved oxygen concentration: 1.7 mg/liter) and severe hypoxia in light [hereafter, submergence (light) and severe hypoxia (light)] (Fig. 1A and fig. S1). Under continuous light without submergence treatment, there was no increase in phosphorylated eIF2α [Fig. 1A, control (light)], indicating that hypoxia upon submergence could induce the phosphorylation of eIF2α and reoxygenation caused the reversal of this induction. We found that eIF2α was phosphorylated in Col-0 in 15 min after transferring from light to dark (Fig. 1A, right panels), but the phosphorylated eIF2α was transiently activated at 15 min under submergence in the dark [hereafter, submergence (dark)] (Fig. 1A, right panels). A recent report showed that in plants transferred to high light intensities after a long duration of darkness (24 hours), reactive oxygen species (ROS) were released from the chloroplast-activated GCN2-mediated phosphorylation of eIF2α (*17*). This raised the possibility that the light-darkness shift and submergence might coordinately fine-tune GCN2-mediated phosphorylation of eIF2α. Thus, we decided to adopt submergence (light), which uncoupled the effects of light-dark shift, to examine GCN2 signaling under submergence. The results showed that the phosphorylation of eIF2α was lost in *gcn2* but restored in two *gcn2* lines with *GCN2* overexpression, *gC9* and *gC14*, under submergence (light) (Fig. 1B and fig. S1). The abundance of eIF2α was not affected under submergence and reoxygenation (Fig. 1B), demonstrating that GCN2 was activated and phosphorylated eIF2α during submergence (light).

To test whether GCN2 signaling affected translation, we examined polysome loading in Col-0, *gcn2*, *gC9*, and *gC14* under submergence (light) (Fig. 1, C to E). Comparing the area of the polysome loading profile curves of the four lines, there was a reduction in polysome loading in Col-0 under submergence (light), indicating general translation repression (Fig. 1, C to E). Compared with Col-0, the reduction of polysome loading was less in *gcn2* but complemented in both *gC9* and *gC14* under submergence (light), revealing that GCN2 is involved in the repression of general translation during submergence (light).

### GCN2-eIF2α signaling is required for submergence

**Fig. 2.**
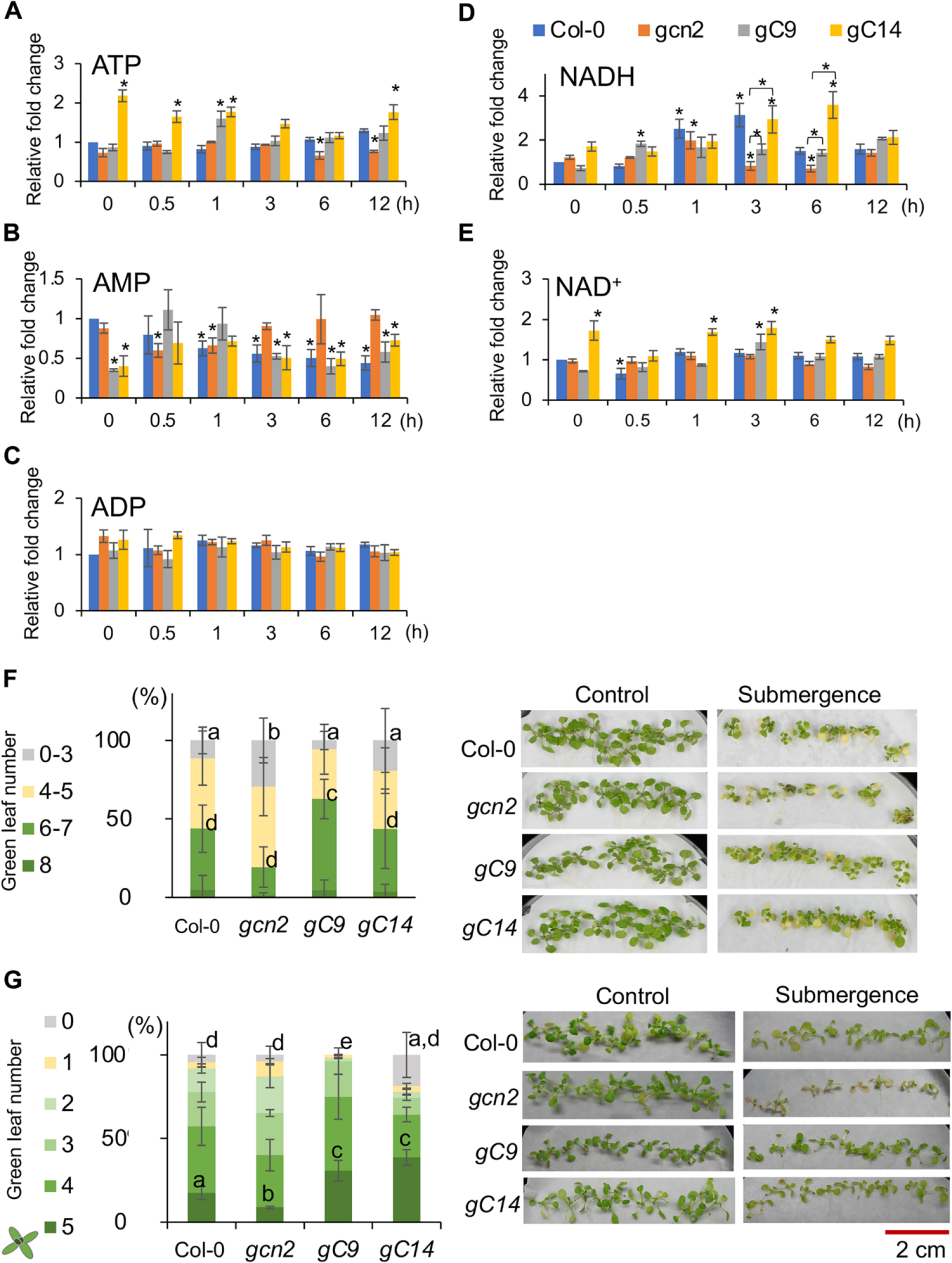
GCN2 is required for tolerance to submergence. (**A** to **E**) The quantification of (A) ATP, (B) ADP, (C) AMP, (D) NADH, and (E) NAD^+^ in the whole seedlings of Col-0, gcn2, and two GCN2 overexpression lines (gcn2 background) under submergence (light). The relative fold change (FC) of each line was normalized with Col-0 under control (time zero). The results represent the mean ± SD of five biological replicates. *P < 0.05. (**F** and **G**) Quantification of survival rate phenotypes (left) and phenotype (right) of 9-day-old Col-0, *gcn2*, and *GCN2* overexpression lines on the third day after (F) 6 days of submergence (light) and (G) 2 days of submergence (dark). The results represent the mean ± SD of three biological replicates. The number of seedlings with different green leaf numbers was calculated into the percentage of total seedlings of each line. The results represent the mean of percentage ± SD of three biological replicates. Different letters represent a significant difference within plant lines determined by Tukey’s test.

We examined the energy-related metabolites and found that the balance of adenosine triphosphate (ATP) and adenosine monophosphate (AMP) under submergence (light) was disrupted in the *gcn2* mutant (Fig. 2, A to E). In Col-0, the level of ATP and ADP (adenosine diphosphate) was maintained but the level of AMP was reduced under submergence (light). In *gcn2*, the level of AMP was transiently reduced in the early stage (0.5 and 1 hour) of submergence, and ATP level was reduced after 6-hour submergence (light). In *gC9* and *gC14*, AMP was reduced, but the level of ATP was higher than that in *gcn2* under submergence (light) (Fig. 2, A to B), implying that GCN2 might mitigate the effects of energy crisis under submergence (light). In addition, the level of NADH [reduced form of nicotinamide adenine dinucleotide (NAD^+^)] increased in all four lines, but the two overexpression lines contained higher levels of NAD^+^ than *gcn2* (Fig. 2, D and E). These results suggested that transgenic lines overexpressing GCN2 could better control energy balance under submergence.

We also examined the tolerance of plants under submergence. The results showed that *gcn2* displayed sensitivity to submergence in both light and dark, which was rescued by overexpressed GCN2 in *gcn2* (Fig. 2, F and G). In addition, we also compared the tolerance of Col-0, *gcn2*, *gC9*, and *gC14* seedlings and mature plants under a short duration (40 and 48 hours) of submergence (dark). In both stages, *gcn2* showed a higher sensitivity to submergence (dark) than the other lines (Fig. 2G and fig. S1), and the two *GCN2* overexpression lines *gC9* and *gC14* had better tolerance to submergence (dark) than Col-0 (Fig. 2G and fig. S1, D and E). These results support the notion that GCN2 is required for submergence tolerance, and transgenic lines overexpressing GCN2 can enhance the tolerance toward submergence.

### Ethylene activates the phosphorylation of eIF2α via a noncanonical pathway under submergence

**Fig. 3.**
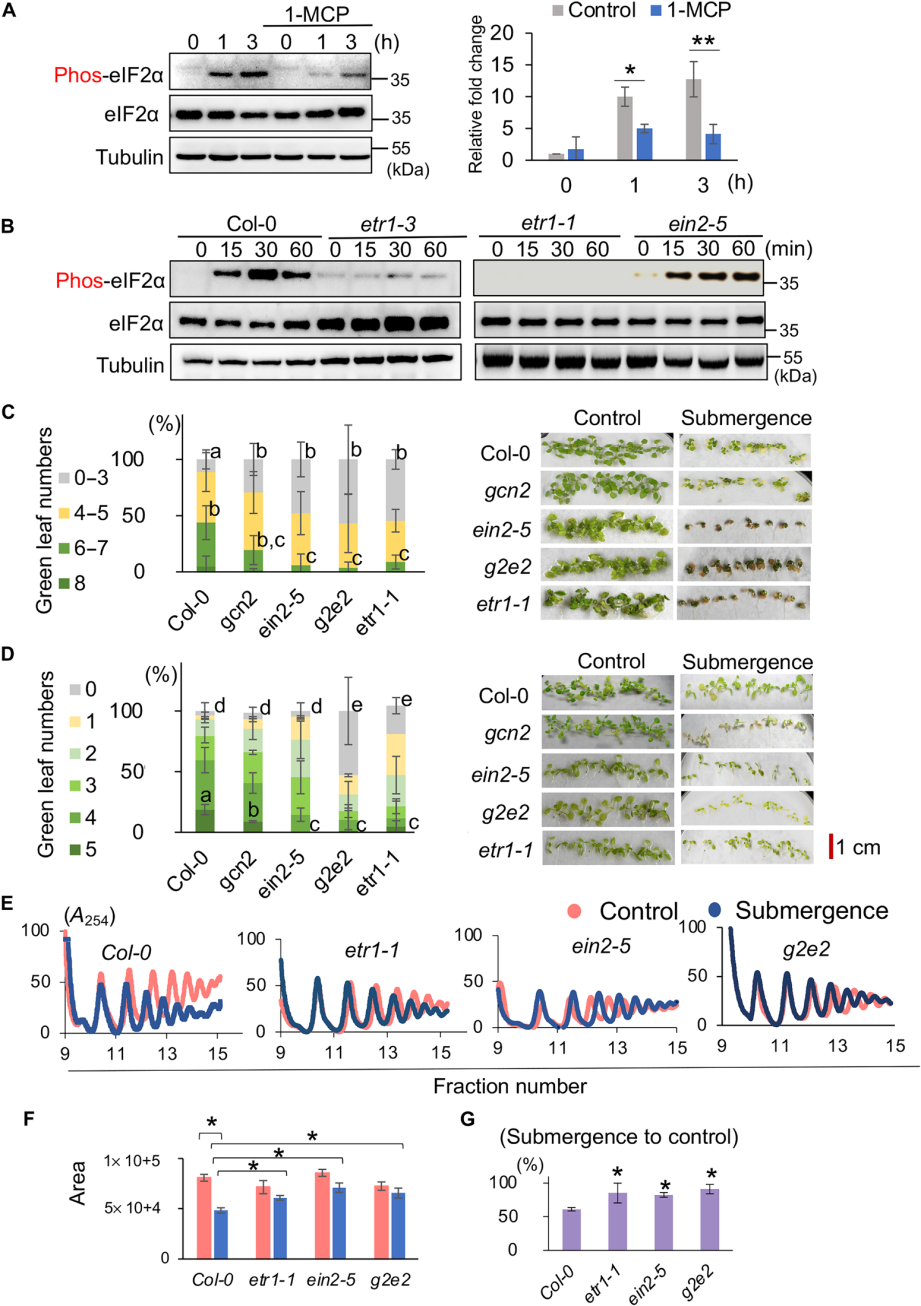
Ethylene modulates general translational repression under submergence (light). (**A**) Western blot showing the levels of phosphorylated and total eIF2α in the whole seedlings of Col-0 under submergence (light) with and without pretreatment with 1-MCP for 2 hours. Tubulin was used as the internal control. Three independent biological repeats showed similar patterns. Bar graph showing the band intensities in the top panel quantified by ImageJ, normalized by the levels of tubulin (bottom) and time zero of the control. (**B**) Western blot showing the levels of phosphorylated and total eIF2α in the whole seedlings of Col-0 and ethylene mutants (*etr1-3*, *etr1-1*, and *ein2-5*) under submergence (light). Tubulin was used as the internal control. Three independent biological repeats showed similar patterns. (**C** and **D**) The quantification of survival rates (left) and phenotypes (right) of 9-day-old Col-0, *gcn2*, *ein2-5*, *gcn2/ein2-5* (*g2e2*), and *etr1-1* on the third day after (C) 6 days of submergence (light) and (D) 2 days of submergence (dark). The number of seedlings with different green leaf numbers was calculated into the percentage of total seedlings of each line. The results represent the mean of percentage ± SD of three biological replicates. Different letters represent a significant difference within plant lines determined by Tukey’s test. (**E**) Polysome profiles of Col-0, *etr1-1*, *ein2-5*, and the *gcn2/ein2-5* double mutant, *g2e2*, under control (red line) and 1-hour submergence (light) (blue line) conditions. Three independent biological repeats showed similar patterns. (**F** and **G**) Bar graphs showing (F) the area of polysome loading profile curves for four lines in (E) under control (red bar) and 1-hour submergence (blue bar) conditions and (G) the ratio of submergence to control areas. The results represent the mean ± SEM of five biological replicates (two-tailed *t* test, *P < 0.05).

It has been reported that GCN2 could be activated by ROS or ACC, which is a direct precursor of ethylene (*15*). Since ROS and ethylene play a major role in hypoxic signaling (*19*, *28*), we decided to examine whether they are upstream signals regulating GCN2 during submergence (light). The results demonstrated that the phosphorylation of eIF2α was transiently activated by 1–part per million (ppm) ethylene (fig. S2A). Under submergence (light), in plants pretreated with 1-methyl cyclopropane (1-MCP), which blocks ethylene binding to ethylene receptors, the phosphorylation level of eIF2α was much lower than that of the control plants (Fig. 3A), and ethephon, which breaks down into ethylene in the water, enhanced the phosphorylation level of eIF2α under submergence (light) (fig. S2B). To further examine whether ROS can activate GCN2 signaling under submergence (light), we transferred plants to the ^1^/_2_ Murashige-Skoog (MS) plated with different ROS inhibitors 2 days before submergence treatment to reduce the production of ROS under submergence (light) (fig. S2C). The results showed no significant difference in eIF2α phosphorylation between antioxidant pretreatment and the control under submergence (light) (fig. S2C). These results imply that ethylene, but not ROS, is involved in the activation of GCN2 under submergence (light).

Next, we examined the phosphorylation of eIF2α under submergence (light) in ethylene mutants. The induction of eIF2α phosphorylation was not detectable in *etr1-1* and *etr1-3* (Fig. 3B and fig. S3A); however, the level of eIF2α phosphorylation in *ein2-5* and *ein3/eil1* was not significantly different from that in Col-0 under submergence (light) (Fig. 3B and fig. S3, A and B). To confirm that the canonical ethylene signaling mediated by EIN2 is activated under submergence (light), we examined the level of EIN3–green fluorescent protein (GFP) in a transgenic line overexpressing EIN3-GFP fusion protein (*29*). EIN3-GFP accumulated during the early stages of submergence (light) (fig. S3C). Furthermore, when examining the *gcn2 ein2-5* double mutant, designated *g2e2*, we found that eIF2α was not phosphorylated in *g2e2* under submergence (light) (fig. S3D), indicating that loss of EIN2 did not turn on other kinases that phosphorylate eIF2α (fig. S3E). Together, these results suggest that ethylene might modulate GCN2 activity via a noncanonical pathway.

To confirm that both GCN2 and EIN2 are required for submergence adaption, we examined the tolerance of Col-0, *gcn2*, *etr1-1*, *ein25*, and *g2e2* under submergence (light and dark) (Fig. 3, C and D). Under both submergence conditions, all four mutants responded similarly that they were more sensitive to submergence (dark and light) than Col-0. The two mutants, *etr1-1* and *g2e2*, displayed the severest damage under submergence (dark and light). Under submergence (light), the survival rates of *etr1-1* and *g2e2* reduced markedly, supporting that the activation of GCN2 and EIN2 by ETR1 plays an essential role in controlling submergence tolerance.

### EIN2 and GCN2 modulate translation dynamics under submergence

**Fig. 4.**
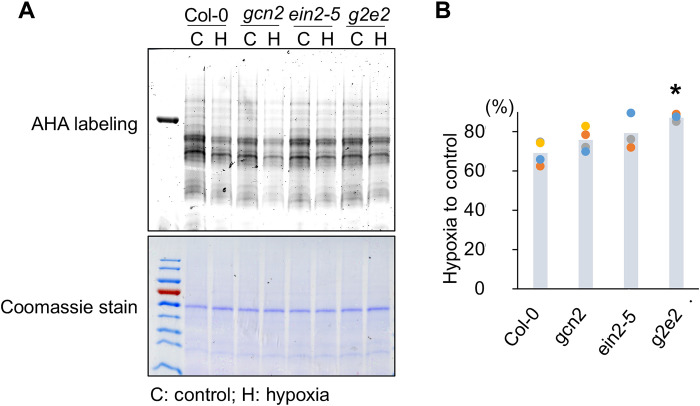
GCN2 and EIN2 modulate the new protein synthesis under hypoxia (3% oxygen, light). (**A**) New synthesis proteins (NSPs) in the whole seedlings of Col-0, *gcn2*, *ein2-5*, and *g2e2* under control and 6-hour hypoxia in the light are visualized by AHA label. The Coomassie-stained gel shows the total protein of four lines under control and 6-hour hypoxia (light). (**B**) Bar graph showing the reduced proportion of NSP under hypoxia in four lines. The quantification of NSP of four lines under control and hypoxia was normalized by the intensity of Coomassie-stained gel. The different color dots represent the results of biological replicates, and the bar represents the mean of biological replicates (two-tailed *t* test, *P < 0.05).

We performed polysome profiling of Col-0, *etr1-1*, *ein2-5*, and *g2e2* under submergence (light) (Fig. 3E). Compared with Col-0, the reduction in polysome loading was significantly lower in *etr1-1* and *g2e2*. Unexpectedly, *ein2-5* displayed a pattern similar to that of *etr1-1* and *g2e2* (Fig. 3, E to G). These results implied that both the canonical EIN2-mediated and noncanonical ethylene-mediated signaling pathways are involved in regulating translation dynamics under submergence. In addition, we also examined the newly synthesized proteins (NSPs) in four lines under hypoxia (3% oxygen) under light [hereafter, hypoxia (light)] (Fig. 4A). The abundance of NSP decreased in Col-0 under hypoxia (light). Although the reduction of NSP was still observed in *gcn2* and *ein2* under hypoxia (light), the reduction of NSP in *g2e2* under hypoxia (light) was much lower (Fig. 4), which supported the notion that EIN2 and GCN2 modulated translation under low oxygen conditions.

**Fig. 5.**
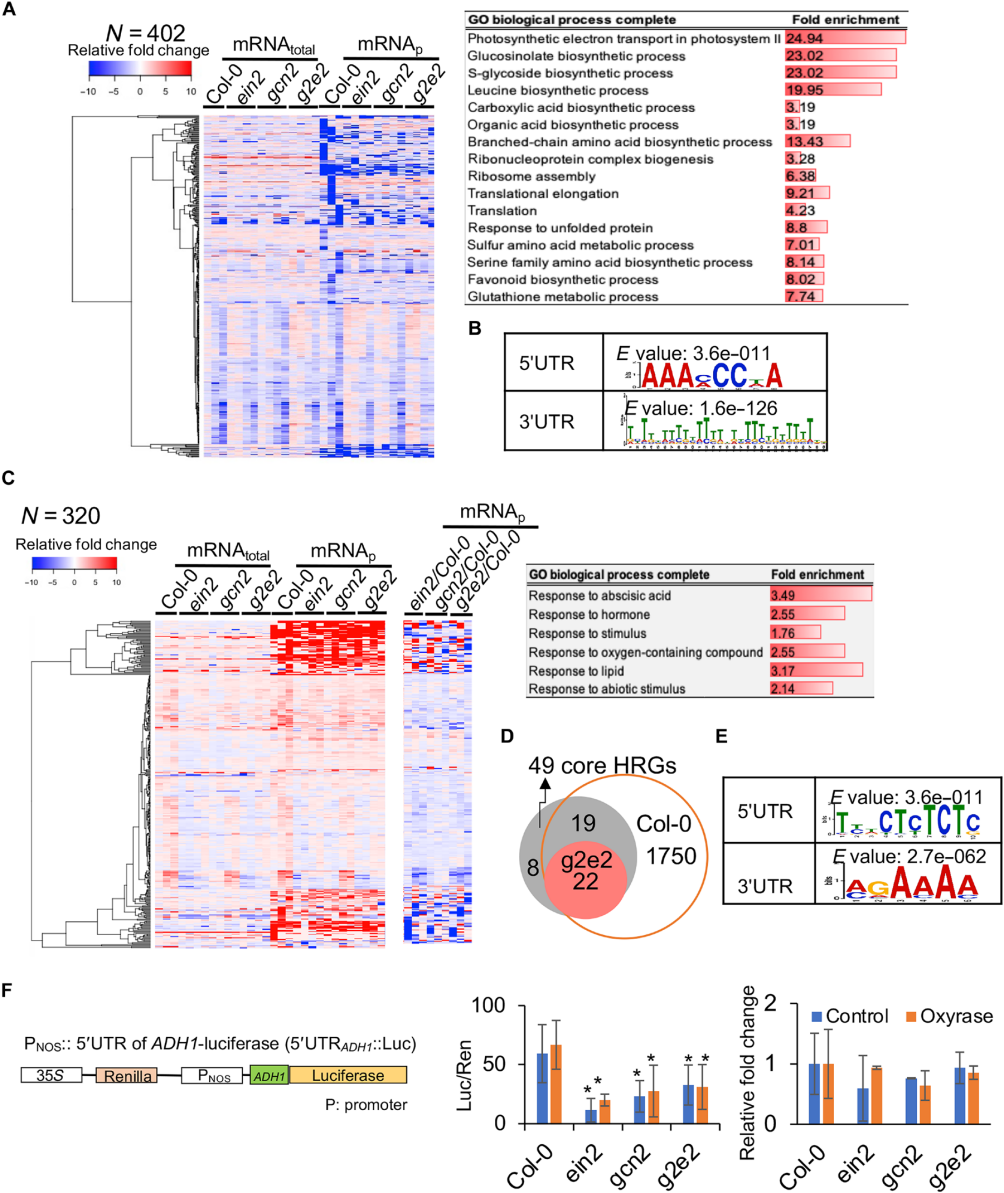
GCN2 and EIN2 modulate translation dynamics under submergence. (**A**) Heatmap and enriched GO terms (*P* < 0.05) for translationally repressed genes in Col-0 that were disrupted in three mutants [*P* < 0.05, mRNA_p_(mutants/Col-0) ≥ 1.5] under submergence (light). (**B**) Sequence motifs in the 5′UTR and 3′UTR of genes that were identified in (A). (**C**) Heatmap and enriched GO terms (*P* < 0.05) for genes with significantly increased levels of translation in Col-0 that were disrupted in three mutants [*P* < 0.05, mRNA_p_(mutants/Col-0) ≤ −1.5] under submergence (light). *N*, total gene number. (**D**) Venn diagram showing that 1790 genes in Col-0 bypassed translational repression, including 41 core HRGs. (**E**) Sequence motifs in the 5′UTR and 3′UTR of genes that were identified in (C). (**F**) Translational activation of the reporter gene (5′UTR of *ADH1*::luc) expressed in Col-0, *ein2*, *gen*2, and *g2e2* protoplasts. Oxyrase (10%) was added to reduce the oxygen level during the incubation. Luciferase activity (left) and relative mRNA level of luciferase (right) were normalized by the activity and mRNA level of Renilla luciferase (Ren), respectively. The data represent the mean ± SD of three biological repeats (two-tailed *t* test, *P < 0.05).

To gain a comprehensive view of the regulation of translation by EIN2 and GCN2 under submergence (light), we generated the transcriptomes and translatomes of Col-0, *gcn2*, *ein2-5* (hereafter, *ein2*), and *g2e2* under submergence (fig. S4). We determined the fold change (FC) in expression during submergence for each gene by calculating the Fragments Per Kilobase of transcript per Million (FPKM) ratio (see Methods for details) between time zero for Col-0 and each of the four lines for both the transcriptome (mRNA_total_) and translatome [translational status (mRNA_p_) and translational efficiency (mRNA_TE_)]. We identified 3552 genes that had a significant FC in mRNA_total_ (*P* < 0.05, |FC| ≥ 2) and 6124 genes that had a significant FC in mRNA_p_ (*P* < 0.05, |FC| ≥ 1.5) (fig. S5A and data S1) in any of the four lines. Among these genes, the translation of 1414 genes was reduced during submergence (light) in Col-0, but translation of 53% of these genes was not reduced in *g2e2* (fig. S5A). We further identified genes whose transcripts showed a significant FC change (*P* < 0.05, FC ≥ 1.5) in translation between Col-0 and the other three lines at any time point. We identified 402 genes, which were involved in photosynthesis, translation, and several biosynthetic processes, which were reduced in Col-0 and showed less reduction in *ein2* or *gcn2*, respectively (Fig. 5A and data S1). Specific mRNA features in the 5′ untranslated region (5′UTR) and 3′UTR were enriched in these mRNAs (Fig. 5B). The mRNA sequence feature in the 3′UTR was previously identified in EIN2 targets and transcripts localized to stress granules (*26*, *27*), implying that these mRNAs might be recruited to P bodies or stress granules by EIN2 under submergence. The translation of mRNAs involved in the process of protein synthesis was not significantly reduced in *gcn2*, whereas the translational reduction of sulfur or organic compound biosynthesis was less severe in *ein2* under submergence (light) (data S1, sheet 4). These results supported our hypothesis that both GCN2 and EIN2 are required to control translational dynamics under submergence (light).

On the other hand, a total of 1790 genes escaped translational repression in Col-0 under submergence (light), of which 320 genes were regulated by EIN2 and GCN2 (data S1 and Fig. 5C). These 320 genes were enriched in the Gene Ontology (GO) terms in response to hormone, lipid, and abiotic stress (data S1 and Fig. 5C). In addition, among 49 core HRGs (*30*), 41 had up-regulated transcript levels and translation in Col-0 under submergence (light) (Fig. 5D and fig. S5B). Of these 41 genes, 22 were modestly lower in *ein2* and *gcn2*, including *ADH1* and *CML38*, and two sequence elements were enriched in the 5′UTRs of the mRNAs (Fig. 5E). We have shown that the 5′UTR of *ADH1* can be recognized by SnRK1-eIFiso4G1 and enhances translation when fused to a reporter protein (*11*). Thus, we examined whether EIN2 and GCN2 can recognize the *ADH1* 5′UTR region and control translation in a protoplast transient assay. We added Oxyrase to reduce the oxygen content when we incubated the protoplasts. The 5′UTR of *ADH1* fused to a luciferase reporter had higher activity in Col-0 under control and Oxyrase treatments than in *ein2*, *gcn2*, and *g2e2* (Fig. 5F); however, the mRNA levels were the same (Fig. 5F, right). These results indicate that EIN2 and GCN2 might also be involved in the translational enhancement of specific mRNAs, and this enhancement mediated by EIN2 and GCN2 might not be only activated in hypoxia. We examined the mRNA levels and translation efficiencies of hypoxia markers in Col-0 and *etr1-1* after 15 min, 1 hour, and 3 hours of submergence (light). The translation efficiencies of *ADH1*, *CML38*, and *CML37* were lower in *etr1-1* than in Col-0 under submergence (light), but the transcript levels were the same as those in Col-0 (fig. S6, A and B). These results indicate that ethylene modulates the translation of specific mRNAs via EIN2 and GCN2 under submergence (light).

### EIN2 and GCN2 regulate transcription during the early stage of submergence (light)

**Fig. 6.**
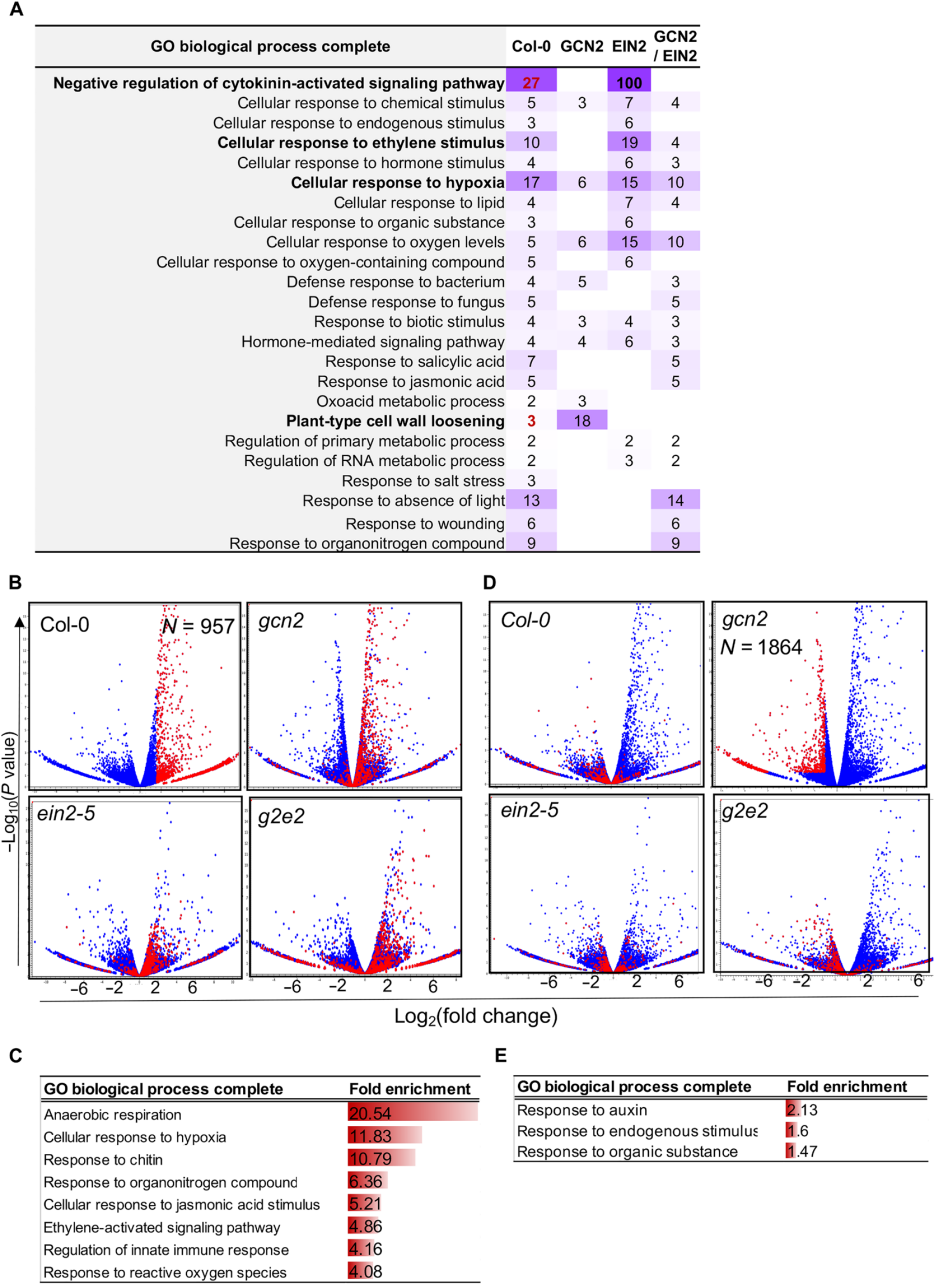
GCN2 and EIN2 modulate transcription under submergence (light). (**A**) Enriched GO terms (*P* < 0.05, fold enrichment > 2) of genes up-regulated under submergence (light) in Col-0 but with significantly lower enrichment in either *gcn2*, *ein2*, and *g2e2*. The number indicated the enriched fold of each GO. (**B**) Scatter plots (volcanic plots) of FC in total mRNA level versus *P* value for four lines after 15 min of submergence (light). Blue dots in each plot represent individual genes, and red dots indicate genes showing significant up-regulation in Col-0. (**C**) Enriched GO terms (*P* < 0.05) of genes whose up-regulation under 15 min of submergence (light) is mediated by EIN2. (**D**) Scatter plots of FC in total mRNA level versus *P* value for four lines after 15 min of submergence (light). Blue dots in each plot represent individual genes, and red dots indicate genes showing significant down-regulation in *gcn2*. (**E**) Enriched GO terms (*P* < 0.05) of genes whose down-regulation after 15 min of submergence (light) is mediated by GCN2.

Submergence (light) for 3 hours markedly changed the gene expression patterns in Col-0, *gcn2*, *ein2*, and *g2e2*. In Col-0, 1205 genes were differentially regulated (*P* < 0.05, FC ≥ 2) under submergence (light). The GO enrichment terms for these differentially expressed genes were similar to those from previous reports (Fig. 6A) and included the response to jasmonic acid (JA), ethylene, salicylic acid, and oxygen levels and innate immune response. Comparing the expression profiles of the four lines, GCN2 mainly affected the expression of plant type cell wall loosening and defense response–related genes (*P* < 0.05, FC ≥ −2) (Fig. 6A), such as *ERF5/6*, *MYB51*, and *MYBL2*, which are also known to be involved in ethylene/JA-mediated responses in *Arabidopsis* (fig. S7A) (*31*, *32*). EIN2 modulated genes involved in ethylene and cytokinin signaling as well as the defense response mediated by ethylene/JA under submergence (light) (Fig. 6A and fig. S7A). Notably, three of five well-known negative regulators of cytokinin signaling—*PUP1* (*AT1G19770*), *KMD1* (*AT1G80440*), and *KMD2* (*AT1G15670*) (*33*)—were induced in Col-0 under submergence (light), but the level of induction was significantly reduced in *ein2* (fig. S7B). These results suggest that ethylene disrupts the cytokinin response and controls plant cell expansion through EIN2 and GCN2 under submergence (light).

In addition, submergence (light) for 15 min significantly enhanced the expression of subsets of genes involved in the responses to ROS and decreased oxygen levels, and the innate immune and ethylene responses (Fig. 6, B and C; fig. S8; and data S1, sheet 6). There was no significant increase in the expression of these genes in *ein2* or *g2e2*. Together, these results indicated that trapped ethylene rapidly activates a primary stress response via EIN2 under submergence (light).

On the other hand, 782 genes showed significant reduction in expression (*P* < 0.05, FC ≤ −2) under submergence (light). The genes related to light response, development, defense response to insects, and the synthesis of secondary metabolites such as glucosinolate and anthocyanin were suppressed (fig. S9A). The expression of genes implicated in glucosinolate biosynthesis, which is chemical defense from cruciferous plants to resist insect herbivores (*34*), was repressed by EIN2 under submergence. These also imply that ethylene controls specific gene abundance to adjust the defense responses under submergence (light) through EIN2 or GCN2.

We found that the expression of auxin response genes was markedly reduced in *gcn2* after 15 min of submergence (light) but recovered to those in the wild type after 1 hour of submergence (light) (Fig. 6, D and E; fig. S9B; and data S1, sheet 7). However, the expression levels of these genes were not significantly different between Col-0 and *ein2* under submergence (light) (Fig. 6D). These results demonstrated that both EIN2 and GCN2 affect the transcription and translation of specific genes under submergence (light).

### Phosphorylation of eIF2α in *Arabidopsis* is required for the translation of specific genes under submergence (light)

**Fig. 7.**
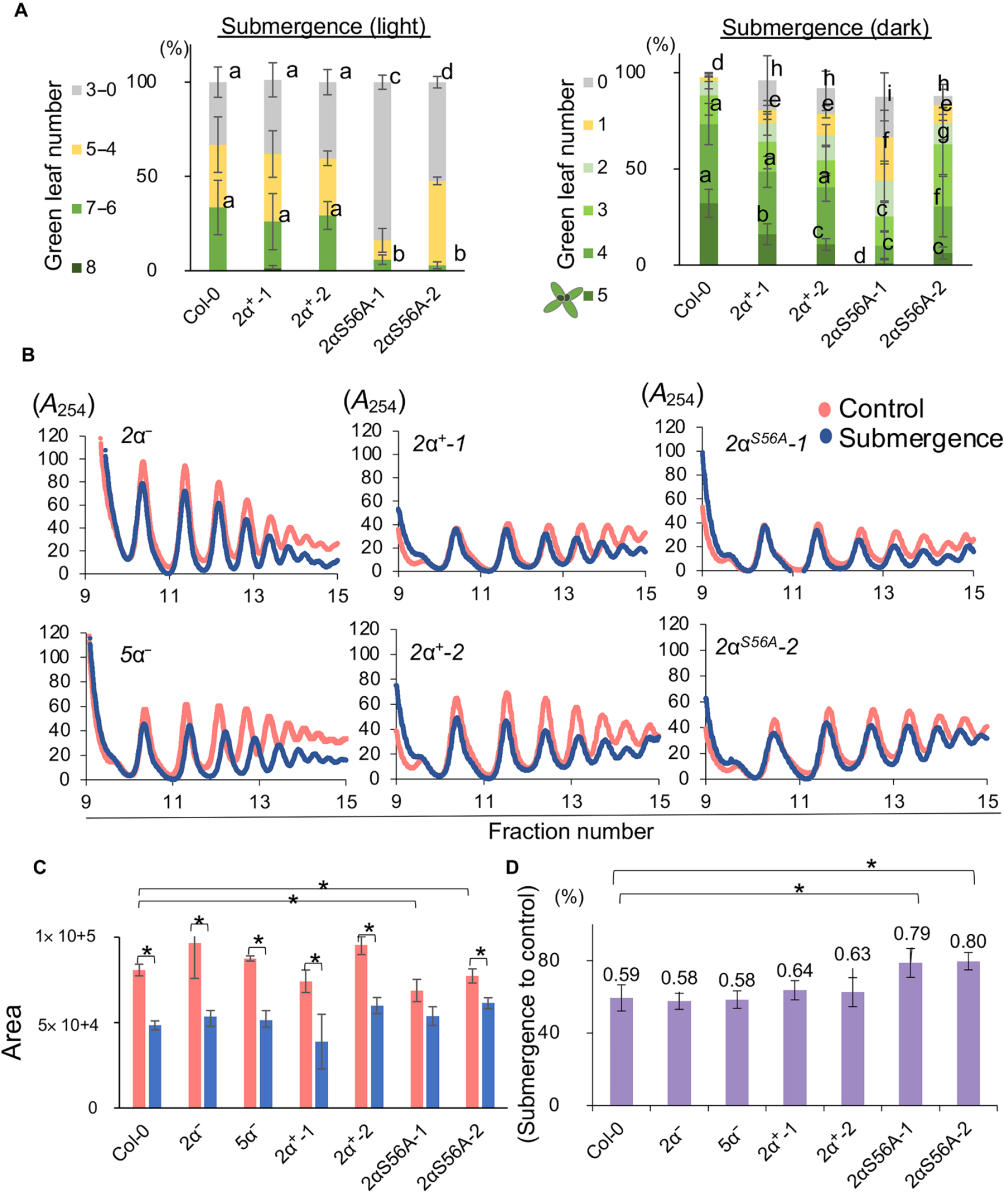
Phosphorylation of eIF2α^S56^ at a conserved site is required for submergence adaption. (**A**) Quantification of green leaf number of 9-day-old Col-0 and *eIF2*α transgenic lines on the third day after (left) 6 days of submergence (light) and (right) 2 days of submergence (dark). The number of seedlings with different green leaf number was calculated into the percentage of total seedlings of each line. The results represent the mean of percentage ± SD of four biological replicates. Different letters represent a significant difference determined by Tukey’s test. (**B**) The polysome profiles of two eIF2α mutants (*2*α*^−^* and *5*α*^−^*), two lines expressing wild-type eIF2α (*2*α*^+^-1* and *2*α*^+^-2*), and two lines expressing S/A-mutated eIF2α (*2*α*^S56A^-1* and *2*α*^S56A^-2*) (in the *2*α*^−^/5*α*^−^* background) under control (red line) and 1-hour submergence (light) (blue line) conditions. Three independent biological repeats showed similar patterns. Bar graph showing (**C**) the area under the polysome profile curves of six lines in (B) under control and 1-hour submergence (light) conditions and (**D**) the ratio of submergence to control area. The results represent the mean ± SEM of five biological replicates (two-tailed *t* test, *P < 0.05).

In *Arabidopsis*, *AT2G40290* and *AT5G05470* encode eIF2α(2g) and eIF2α(5g), respectively, which can be phosphorylated at Ser^56^ by GCN2 in vitro (*35*). To test the role of the phosphorylation of eIF2α under submergence (light), we generated transgenic lines containing various mutations of eIF2α (Table 1). A homozygous T-DNA insertion line of *eIF2*α(*5g*), designated *5*α*^−^*, showed no differences from Col-0 and was able to produce seeds (fig. S10). However, a homozygous T-DNA insertion line of *eIF2*α(*2g*), designated *2*α*^−^*, was small and lost the ability to produced seeds (fig. S10). In *2*α*^−^* lines, almost no eIF2α protein accumulated (fig. S10C), implying that the dosage of eIF2α is critical for plant development and that eIF2α(2g) plays a major role during development. We next constructed transgenic lines expressing similar amounts of wild-type eIF2α (2α^+^) and S/A-mutated eIF2α (2α^S56A^) in the *eif2*α(*2g*)/*eif2*α(*5g*) background (fig. S11, A and B; see Methods for details). These transgenic lines were evaluated for survival ability and polysome loading under submergence. The *2*α*^+^* lines and *2*α*^S56A^* lines were not different from Col-0 in terms of development, but the *2*α*^S56A^* lines were more sensitive to submergence (light and dark) (Fig. 7A and fig. S11, C and D). Similar to Col-0, polysome loading under submergence (light) was reduced in two independent *2*α*^+^* lines (Fig. 7, B to D). In contrast, there was no significant reduction in polysome loading in two independent *2*α*^S56A^* lines under submergence (light). However, compared with Col-0, polysome loading under normoxia was lower in the *2*α*^S56A^* lines (Fig. 7, B to D).

**Table 1.** *eIF2*α mutant lines.

**Line**	**Genotype**	**Description**
2α^−^	*eif2*α(2g)	T-DNA insertion line of *eIF2*α (AT2G40290)
5α^−^	*eif2*α(5g)	T-DNA insertion line of *eIF2*α (AT5G05470)
2α^+^-1	*eIF2*α(2g)	Transgenic line containing wild-type *eIF2*α (AT2G40290) in *eif2*α(2g)/*eif2*α(5g) background
2α^+^-2	*eIF2*α(2g)	
2α^S56A^-1	*eIF2*α(2g S56A)	Transgenic line containing S56A mutation of *eIF2*α (AT2G40290) in *eif2*α(2g)/*eif2*α(5g) background
2α^S56A^-2	*eIF2*α(2g S56A)	

**Fig. 8.**
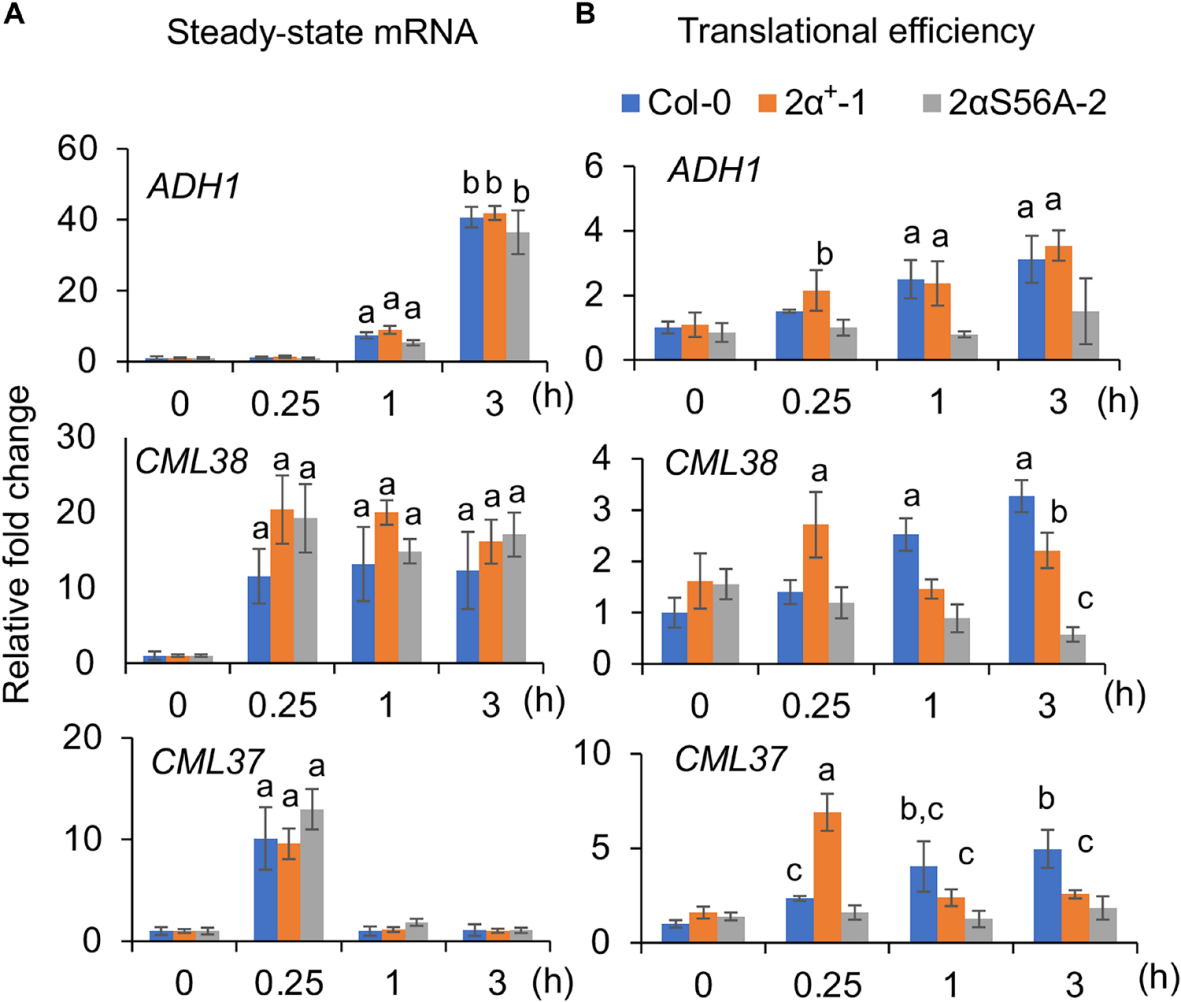
Lack of phosphorylated eIF2αS56 disrupts the translation of specific mRNAs under submergence (light). (**A**) The transcript levels of HRGs in the whole seedlings of Col-0 and *eif2*α lines were quantified by qPCR, and tubulin was used as the internal control. The relative FC of each line was normalized with Col-0 under control (time zero). Values represent the mean of relative FC ± SD (four biological replicates). Different letters represent a significant difference determined by Student’s *t* test. (**B**) The translational status of HRGs in the whole seedlings of Col-0 and *eif2*α lines was quantified by qPCR, and the mRNA expression level at each indicated time point was used as an internal control. The relative FC of each line was normalized with Col-0 under control (time zero). Values represent the mean of relative FC ± SD (four biological replicates). Different letters represent a significant difference within plant lines determined by Tukey’s test.

To determine whether phosphorylated eIF2α could affect the translation of specific mRNAs, we quantified the translational efficiencies of *ADH1*, *CML38*, and *CML37* in the Col-0, *2*α*^+^*, and *2*α*^S56A^* lines under submergence (light) (Fig. 8). The results showed that the translation of *ADH1*, *CML38*, and *CML37* was affected in the *2*α*^S56A^* lines to an extent similar to that observed in *gcn2* and *etr1-1* (figs. S5 and S6). These results demonstrated that phosphorylation of eIF2α is required for the translational regulation mediated by GCN2 under submergence (light).

## DISCUSSION

### Ethylene modulates translation and transcription via EIN2 and GCN2 in *Arabidopsis* under submergence

In mammals and yeast, both target of rapamycin (TOR) and protein kinase RNA-like endoplasmic reticulum kinase (PERK) act as signal hubs repressing translation to conserve energy and ensure the production of proteins required under stress (*36*, *37*). In plants, we found that the SnRK1-eIFiso4G1 signaling relay can enhance the translation of specific mRNAs, thus bypassing translational repression under submergence (*11*). However, how plant cells sense a decrease in oxygen and quickly reduce translation is still unclear. Here, we showed that GCN2 phosphorylates eIF2α after 15 min of submergence (light) (Fig. 1A). Under submergence (light), phosphorylation of eIF2α is maintained for at least 3 hours but quickly decreases when plants are removed from submergence (Fig. 1A). Analyses of translational profiles in *gcn2* and *2*α*^S56A^* (Figs. 1, B to E, and 7, B to D) showed that the GCN2-eIF2α signaling is triggered during submergence to control repression of general translation and the translation of specific mRNAs. In addition, we also confirmed that GCN2 and phosphorylated eIF2α are critical for the tolerance of seedlings to submergence (Figs. 2 and 7A). Transgenic lines overexpressing GCN2 showed enhanced tolerance to submergence and retained higher ATP and NAD^+^ levels (Fig. 2), indicating that GCN2 is critical for energy balance under submergence.

GCN2 is activated by ethylene under normal and submergence (light) conditions (*15*) (Figs. 1 and 3 and figs. S1 and S2). Under submergence (light), plants would not suffer from hypoxia immediately, since the oxygen concentration in the petiole was not decreased markedly (*38*). On the other hand, the rapid increase in ethylene under submergence triggered prepriming mechanism to prepare for hypoxia upon submergence (*22*, *23*), indicating that early activated GCN2-eIF2α under submergence (light) is a previously unidentified part of the prepriming mechanism triggered by ethylene for subsequence hypoxia. The activity of GCN2 was disrupted in *etr1-1* and *etr1-3*, but not in *ein2* (Fig. 3B) or *ein3/eil1* (fig. S3B), under submergence (light). These results suggested that the binding of ethylene to ETR1 triggers a noncanonical pathway that activates GCN2 under submergence. The noncanonical ethylene pathway (EIN2-independent pathway) was proposed to control stress responses under abiotic stress (*39*), but the exact signaling transduction steps for the pathway are not clear. Here, our data demonstrated that GCN2-eIF2α is a novel ethylene signal cascade to control stress response, through translational adjustment (Fig. 5).

Strikingly, EIN2 was also involved in general translation repression under submergence (light) (Figs. 3 to 5). Through transcriptome and translatome analysis, we found that both EIN2 and GCN2 were required for the translation repression and transcription of specific genes under submergence (light) (Figs. 5 and 6 and figs. S7 to S9). EIN2 plays a major role in modulating the expression of genes involved in ethylene and cytokinin signaling under submergence (light) (Fig. 6, A to C, and figs. S7 and S8). GCN2 can enhance the expression of genes involved in plant-type cell wall loosening (Fig. 6A), indicating that either EIN2 or GCN2 is required for submergence adaption. Therefore, we propose that *Arabidopsis* plants quickly sense submergence and prepare for hypoxia upon submergence through activation of both EIN2 and GCN2 by the entrapped ethylene during submergence; activation of these proteins ensures that proteins required for stress responses under submergence are produced (fig. S12).

### Ethylene is the primary activator of GCN2 under submergence (light) but might not be the only upstream regulator in *Arabidopsis* under oxygen deprivation

The upstream regulators of GCN2 in plants remain elusive, especially the true biochemical signal. GCN2 activity is activated by several agents, including phytohormones, UV, herbicides, ROS, and pathogens (*15*, *16*). However, it is not clear whether these agents regulate GCN2 in nature. Here, we found that GCN2 was activated by entrapped ethylene upon submergence as well as severe hypoxia in the gas jar under light, from which the ethylene is easily released out of plants (Figs. 1 and 3 and figs. S1 and S2), implying that ethylene would not be the only factor modulating GCN2 function under submergence. On the other hand, GCN2 was constantly activated when plants were transferred from light to dark, but its activity was only transiently activated within 15 min when plants were transferred from light to submergence in the dark (Fig. 1A). GCN2-eIF2α was required for submergence adaptation in the dark (Figs. 2G and 7A and figs. S1 and S11), and overexpression of GCN2 enhanced the submergence tolerance in the dark (fig. S1). Recent studies have revealed that high levels of ROS released by chloroplasts under herbicide treatment activate GCN2 activity and that the hypoxia and ethylene responses are enhanced in *gcn2* under herbicide treatment (*15*, *17*). Thus, it is highly possible that the light-dark shift would trigger a high dosage of ROS and coordinate with entrapped ethylene to activate GCN2 under submergence in the dark. It will be interesting to investigate how ETR1 regulates GCN2 activity under submergence and dissect the mechanism through which plants adjust GCN2 activity so quickly under submergence in the dark.

### The function of GCN2-eIF2α is conserved in eukaryotic cells

The function of phosphorylated eIF2α has remained elusive in plants. The composition of the eIF2B complex is not similar to that in yeast and mammals (*40*), and the affinity of GTP and GDP for eIF2 in plants is not as high as that in yeast and mammals (*18*, *40*). These observations raise the question of whether the phosphorylation of eIF2α is related to translational repression like it is in yeast and mammals. We showed that phosphorylated eIF2α is involved in general translation repression and affects the translation of specific mRNAs under submergence (Figs. 7 and 8), indicating that plant GCN2-eIF2α still has some conserved functions in translational regulation.

However, transgenic lines expressing *eIF2*α*^S56A^* did not show the same degree of effects on polysome loading as *gcn2* under submergence (Figs. 1 and 7), implying that eIF2α^S56^ might not be the only phosphorylation site recognized by GCN2 or that other proteins participate in GCN2 signaling under submergence. On the other hand, GCN2 might have other interactors that determine its function under submergence, such as factors that regulate GCN2 under cold stress in *Arabidopsis* and amino acid starvation in yeast (*41*, *42*).

### GCN2-eIF2α and EIN2 might couple with UBP1C to regulate dynamic translation under submergence

We found that the 3′UTRs of mRNAs whose translational repression was mediated by GCN2 and EIN2 under submergence shared T-rich elements (U-rich in mRNA) (Fig. 5B). This feature was also identified in the mRNAs sent to stress granules, which is mediated by UBP1C, or P bodies, which is mediated by EIN2 (*8*, *26*, *27*). These results imply that EIN2 and GCN2 might couple with UBP1C to confer translational repression under submergence. These also raise the possibility that EIN2 and phosphorylated eIF2α by GCN2 coordinately modulate translational repression under submergence. It will be interesting to examine whether phosphorylated eIF2α by GCN2 is also involved in the foci formation, like P bodies, with EIN2 under submergence.

Together, our results reveal that a noncanonical ethylene signaling pathway triggers GCN2/eIF2α to modulate translation and transcription in *Arabidopsis* under submergence. These findings provide a new perspective on how eukaryotic cells regulate translation under abiotic stress.

## METHODS

### Plant materials and hypoxia/anoxia treatments

The T-DNA insertion line of *gcn2* (GK-862-B02) was obtained from the Nottingham Arabidopsis Stock Centre, UK, and the ethylene signal–related mutants [*ein2-5*, *etr1-1*(CS237), *etr1-3*(CS3070), and *ein3/eil1*] and two *eif2*α mutants [*eif2*α(*2g*) (SALK_025426) and *eif2*α(*5g*) (SALK_065838) lines] were obtained from the Arabidopsis Biological Resource Center, Ohio State University, USA. The homozygous examination of ethylene signaling–related mutants was performed according to An *et al.* (*43*). The transgenic lines *gC9* and *gC14* overexpressing *GCN2* in the *gcn2* background were generated by T-DNA–mediated transformation of *gcn2* with *pEarleygate 101* containing wild-type *GCN2*. Because the double mutant of *eif2*α is lethal, transgenic lines expressing the wild-type *eif2*α genes, p*eIF2*α(2g)::*eIF2*α(2g) and p*eIF2*α(5g)::*eIF2*α(5g), and *eif2*α genes with the S56A substitution, p*eIF2*α(2g)::*eIF2*α(2g^S56A^), were generated by T-DNA–mediated transformation with *eif2*α(*2g*)^+/−^/*eif2*α(*5g*)^−/−^, and then independent transgenic lines in a homozygous background, *eif2*α(*2g*)^−/−^ or *eif2*α(*5g*)^−/−^, were selected. The independent transgenic lines were characterized in the T2 or T3 generation. The *ein2/gcn2* double mutant was created by crossing *ein2-5* to *gcn2*. All the transgenic lines and mutants used in this study are listed in table S1.

Germination and growth of *Arabidopsis* plants were as described previously (*44*). Seeds were sterilized with 0.5% sodium hypochlorite for 10 min, washed at least three times with sterilized water, and then incubated with sterilized water at 4°C for 3 days to achieve uniform germination. The seedlings were transferred into new ^1^/_2_ MS medium plates or ^1^/_2_ MS medium plates with 0.5 mM ascorbate sodium and 100 nM diphenyleneiodonium (DPI) 2 days before submergence.

Nine-day-old seedlings grown in a growth chamber under a 16-hour light (81 μmol m^−2^ s^−1^; at 22°C)/8-hour dark (at 18°C) cycle were submerged 2 hours after the start of the photoperiod in ^1^/_2_ MS medium that was prebubbled with 3% oxygen for 1 hour (the final dissolved oxygen concentration: 1.7 mg/liter) in the light (81 μmol m^−2^ s^−1^). ^1^/_2_ MS medium was kept bubbled with 3% oxygen when seedlings were submerged. Seedlings kept at room temperature in the light were used as the control. For severe hypoxia treatment, 9-day-old seedlings on plates were incubated in the gas jar with 99% nitrogen in the light, and the whole seedlings were harvested at the indicated time.

For examination of the submergence tolerance of seedling, 9-day-old seedlings on plates were completely submerged in sterilized ^1^/_2_ MS medium in the dark for 48 hours or in sterilized water with ampicillin (100 μg/ml; Sigma-Aldrich) and 1:4000 (v/v) EC-Oxyrase (Sigma-Aldrich) in the constant light (81 μmol m^−2^ s^−1^; at 22°C) for 6 days. After submergence treatment, plants were transferred to the filter paper with 2.5 ml of sterilized water and put back into a growth chamber under a 16-hour light (81 μmol m^−2^ s^−1^; at 22°C)/8-hour dark (at 18°C) cycle, and the number of damaged or chlorotic leaves was counted after 3 days of recovery. Thirty plants (per line) were treated in each replicate. We found that 48 hours of submergence (dark) and 6 days of submergence (light) caused damage to 50% of Col-0 seedlings. Thus, 48 hours of submergence (dark) and 6 days of submergence (light) were used to examine the tolerance of plants.

For submergence tolerance examination of mature plants, 5-week-old potted plants were placed into distilled water at a depth of at least 5 cm from the water surface in the dark at 22°C. To prevent the pot and medium from floating during submergence, five glass beads (1.6-cm diameter) were loaded into the bottom of pots before plants were transplanted. After 40 to 48 hours of submergence treatment, plants were put back into a growth chamber under a 9-hour light (81 μmol m^−2^ s^−1^; at 22°C)/15-hour dark (at 18°C) cycle, and the plants that were still alive 2 weeks after submergence were counted as surviving. Nine plants (per line) were treated in each replicate.

### 2′,7′-Dichlorofluorescein diacetate stain

To specifically detect ROS, fluorescent staining with 2′,7′-dichlorofluorescein diacetate (H_2_DCFDA; Sigma-Aldrich) was performed. Roots of the untreated or 1-hour submergence–treated 9-day-old seedlings were incubated in the dark with 15 μM H_2_DCFDA for 10 min. After triple washing with double-distilled water, the seedlings were examined under a confocal microscope (Zeiss LSM 780). The fluorescent intensity was quantified by the built-in microscope software.

### 1-MCP, ethylene, and ethephon treatments

For 1-MCP treatment, five plates of 9-day-old seedlings (total of 40 seedlings) were placed in a gas chamber, which was filled with 1-MCP (LytoFresh) mixed with air, for 2 hours. Then, the plants were treated with submergence and harvested at the indicated times to extract proteins. For ethylene treatment, two plates of 9-day-old seedlings (total of 20 seedlings) were placed in a gas chamber, which was filled with 1-ppm ethylene mixed with air, and then the plants were harvested at the indicated times to extract proteins. For ethephon treatment, 2-chloro ethylphosphonic acid (Ethephon, Yuan Mei Biotech, Taiwan) was added into the ^1^/_2_ MS medium, which was prebubbled with 3% oxygen for 1 hour, to reach the final concentration, 100 μΜ ethephon. Then, the seedlings were submerged into the ^1^/_2_ MS medium with or without ethephon and harvested at the indicated times to extract proteins.

### RNA extraction and polysome fractionation

Nine-day-old seedlings with or without submergence treatment were harvested to extract RNA. Total RNA and RNA loaded on polysomes were isolated and quantified as previously described (*45*). Because the polysome profiles were altered in mutants under normal conditions, to precisely compare the amount of RNA loaded on polysomes under submergence and control conditions, the base of *A*_254_ (absorbance at 254 nm) line between the peaks of 80*S* and monosome in each plant line under different treatments was aligned to zero. Then, the areas under the polysome profile curves and the ratio of areas of each plant line under control and treatment were calculated. For RNA sequencing, the RNA fraction was separated into two parts: polysome-free RNA and polysome-bound RNA to quantify the mRNAs specifically loaded on the polysome.

### Detection of new synthesis proteins

Before labeling the NSPs, 9-day-old seedlings were transferred to filter paper with ^1^/_2_ MS medium for 1 hour to reduce the wounding response. Then, nine plants were transferred to the filter paper soaked with 1.8 ml of ^1^/_2_ MS medium containing 100 μM l-azidohomoalanine (Click-IT AHA, Invitrogen) and treated with 3% oxygen or air under light for 6 hours. To detect the AHA-labeled proteins, the Click-IT Tetramethylrhodamine Protein Analysis Detection Kit (Invitrogen) was applied according to the instructions. Briefly, 60 μg of total proteins extracted by extraction buffer [1% SDS, 50 mM tris-HCl (pH 8.0), 1:250 (v/v) proteinase inhibitor cocktail for plant cell lysate (Sigma-Aldrich), and 2 μΜ phenylmethylsulfonyl fluoride (Sigma-Aldrich)] were mixed with the 0.4× standard volume of Click-iT reaction buffers and then precipitated by methanol and chloroform. The precipitated protein was suspended by 1× sample loading buffer and denatured at 70°C for 10 min. The denatured mixture was loaded into the 4 to 20% protein gel (Novex, Invitrogen), and the fluorescence was detected by Sapphire Biomolecular Imager (Azure Biosystems) with excitation at 520 nm and emission at 565 nm. The fluorescence intensity was quantified by ImageJ and normalized by Coomassie blue stain.

### RNA sequencing and data analysis

For transcriptome and translatome analysis, two independent biological repeats of 9-day-old seedlings of Col-0 and three mutants—*ein2-5*, *gcn2*, and *g2e2*—at the same developmental stage were harvested after 15 min, 1 hour, and 3 hours of submergence in the light and immediately frozen in liquid nitrogen for polysome fractionation and RNA extraction.

Paired-end complementary DNA (cDNA) libraries for total RNA and polysome RNA were constructed and sequenced on an Illumina HiSeq2500 platform at the High Throughput Genomics Core of Academia Sinica, Taiwan. For sequencing data mapping, a reference transcript dataset [The Arabidopsis Information Resource (TAIR) 10 cDNA update] with 41,672 annotated protein-coding gene sequences was retrieved from TAIR. The raw reads were preprocessed and normalized using CLC Genomics Workbench 11 (Qiagen). The raw reads in each batch were analyzed to give the expression ratio of genes between submergence and control conditions at indicated time points for the total RNA and polysome RNA samples using the Digital Gene Expression empirical analysis tool of CLC Genomics workbench 11. For comparison between Col-0 and mutants, the FPKM values were normalized by the 75th percentile relative FPKM value for total RNA and polysome RNA within the treatment and control. To investigate the correlation of total mRNA (mRNA_total_), translational status (mRNA_P_), and translational efficiency (mRNA_TE_), the genes in Col-0, *ein2*, *gcn2*, and *g2e2* with tagwise dispersions *P* < 0.05, |FC| ≥ 2 for mRNA_total_ and tagwise dispersions *P* < 0.05, |FC| ≥ 1.5 for mRNA_P_ or mRNA_TE_ were selected. The genes that showed significant difference (*P* < 0.05, |FC| ≥ 2 for mRNA_total_ or *P* < 0.05, |FC| ≥ 1.5 for mRNA_P_) between Col-0 with one of mutants were further clustered. The GO categories enriched in each cluster were determined using the GO term enrichment tool of TAIR (test type: Fisher’s exact test; correction: Bonferroni correction for multiple testing).

### Quantitative polymerase chain reaction

Reverse transcription and quantitative polymerase chain reaction (qPCR) was performed as previously described (*44*). The primers for the genes analyzed are listed in table S2. The relative expression levels were calculated as the ratio between the mRNA levels in the sample at specific time points and the levels in the sample at time zero. For quantification of mRNA levels in the polysome fraction, RNA (1 μg) was reverse-transcribed. The primers used are listed in table S2. The translation efficiency was calculated as mRNA_P_/mRNA_total_, using the mRNA_P_ and mRNA_total_ values from the same biological experiment for each line.

### 5′UTR and 3′UTR analysis

The 5′UTR and 3′UTR sequences of selected genes were downloaded from TAIR10. The MEME program with the default options (*46*) was used to find shared functional motifs in the 5′UTRs and 3′UTRs of the EIN2 and GCN2 targets. *E* values (<0.05) were used to estimate the probability of finding a motif by chance.

### Transient assays

For transient reporter assays, constructs with the nopaline synthase (NOS) promoter driving the expression of the 5′UTR of *ADH1* fused with the firefly luciferase (Luc) coding region were made using the pGreenll-0800 Luc plasmid (*47*), which also contains the 35*S* promoter driving Renilla luciferase (Ren) expression. The luciferase activity assays were performed following the instructions of the Dual Luciferase Reporter Assay System (Promega). The Luc/Ren ratio of each sample was calculated. For low oxygen treatment, 1:10 (v/v) Oxyrase (Sigma-Aldrich) was added after protoplast transformation and protoplasts were incubated overnight. For the quantification of reporter expression, reverse transcription and quantitative reverse transcription PCR were performed as previously described (*44*). The primers for the genes analyzed are listed in table S2.

### Western blot analysis

The protein extraction process was according to (*48*). The Western blots were hybridized with GFP (titer 1:3000; Roche), tubulin (titer 1:10,000; Sigma-Aldrich), phospho-eIF2α (Ser^51^) (titer 1:2000; Abcam), and eIF2α (titer 1:2000) antibodies. The eIF2α antibody was raised against a specific peptide derived from the deduced amino acid sequence (MILFSELSRRRIRS). The band intensities were quantified by ImageJ software.

### ATP, ADP, AMP, NAD^+^, and NADH assays

Nine-day-old seedlings (80 mg) subjected to different durations of submergence were collected, and ATP, ADP, AMP, NAD^+^, and NADH were quantified by liquid chromatography–MS/MS as described previously (*48*).

### Accession numbers

The RNA sequence data have been deposited in the National Center for Biotechnology Information (NCBI) Gene Expression Omnibus database with the dataset identifier GSE149414.

The gene accession numbers are as follows: *GCN2* (AT3G59410), *eIF2*α (AT2G40290, AT5G05470), *ADH1* (AT1G77120), *CML38* (AT4G33070), *CML37* (AT5G42380), *ETR1* (AT1G66340), *EIN2* (AT5G03280), *EIN3* (AT3G20770), *EIL1* (AT2G27050), *ERF5* (AT5G47230), *ERF6* (AT4G17490), *MYB51* (AT1G18570), and *MYBL2* (AT1G71030).

## Notes

View/request a protocol for this paper from *Bio-protocol*.
