# Supplementary material for: Ethylene modulates translation dynamics in Arabidopsis under submergence via GCN2 and EIN2
Source: Sci Adv. 2022 Jun 3;8(22):eabm7863. doi: 10.1126/sciadv.abm7863 (PMC9166634; doi:10.1126/sciadv.abm7863)
Supplement: Supplementary file 1 — Figs. S1 to S12 [file sciadv.abm7863_sm.pdf]

Science Advances
AAAS

Supplementary Materials for

Ethylene modulates translation dynamics in *Arabidopsis* under submergence via GCN2 and EIN2

Hsing-Yi Cho *et al.*

Corresponding author: Ming-Che Shih, mcshih@gate.sinica.edu.tw

Sci. Adv. **8**, eabm7863 (2022)
DOI: 10.1126/sciadv.abm7863

The PDF file includes:

Figs. S1 to S12

Other Supplementary Material for this manuscript includes the following:

Tables S1 and S2
Data S1

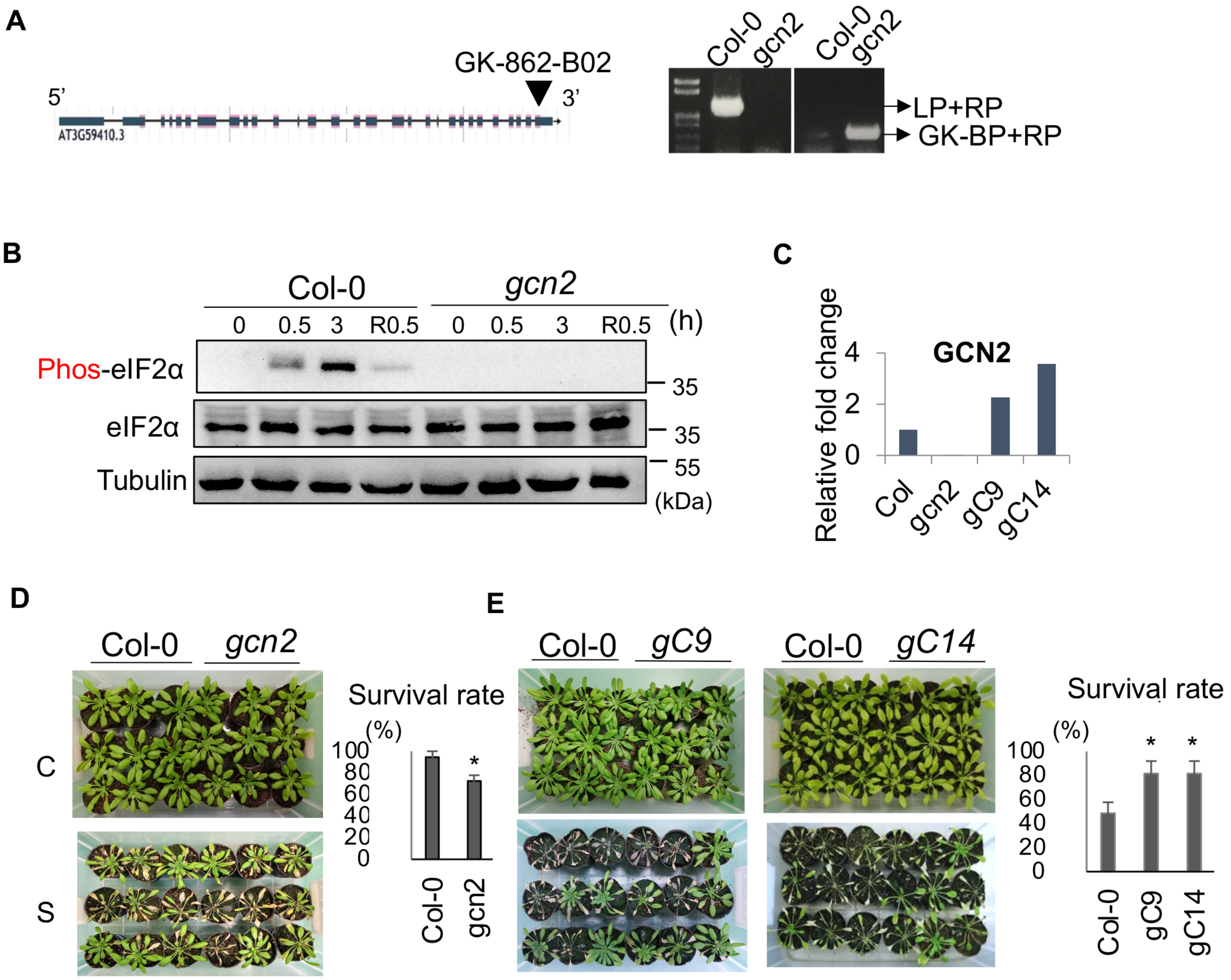

Figure S1. Isolation of the GCN2 knockout and overexpression lines of Arabidopsis. (A) The gene structure of GCN2 and verification of the genotype of gcn2. (Upper panel) Diagram of the gene structure of GCN2 (adopted from TAIR) and the location of the T-DNA insertion in gcn2 (GK862-B02). Squares and lines indicate the exons and introns, respectively. The triangle indicates the location of the T-DNA insertion. (Lower panel) Genotyping of gcn2. Genomic DNA of Col-0 and gcn2 was amplified with GK BP-8474 (GK-BP) and RP to detect the T-DNA insertion, and with RP and LP primers to detect the wild-type allele. (B) Western blots showing the phosphorylation profile of eIF2α in the whole seedlings of Col-0 and gcn2 under severe hypoxia (light). Tubulin was used as the internal control. R: Recovery. Three independent biological repeats showed a similar pattern. (C) The transcript levels of GCN2 in the whole seedling of Col-0 and gcn2 mutants were quantified by qPCR, and tubulin was used as the internal control. (D) The phenotypes (left) and quantification of survival rates (right) of 5-week-old Col-0 and gcn2 plants after 40 h of submergence. (E) The phenotypes (left) and quantification of survival rates (right) of 5-week-old Col-0 and GCN2 overexpression lines (gcn2 background) after 48 h of submergence under dark. The results represent the mean ± SD of three biological replicates. *, $P < 0.05$.

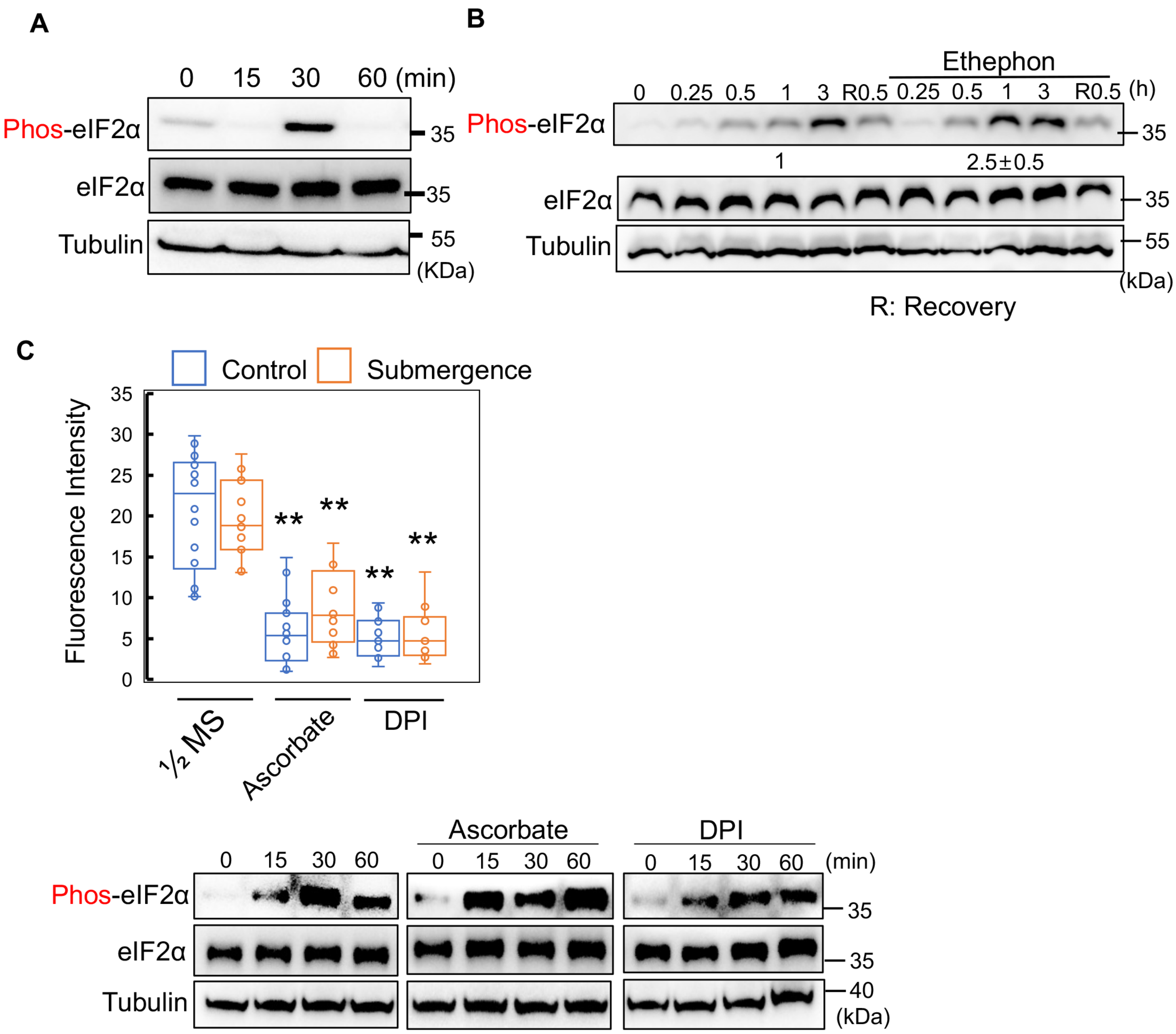

Figure S2. The phosphorylation of eIF2α is enhanced by ethylene but not ROS under submergence (light). (A) Western blot showing that 1 ppm ethylene transiently activated the phosphorylation of eIF2α in the whole seedlings of Col-0. Tubulin was used as the internal control. Three independent biological repeats showed similar patterns. (B)Western blot showing that phosphorylation of eIF2α was induced in the whole seedlings of Col-0 on ½ MS plates and enhanced by ethephon pretreatment under submergence (light). Tubulin was used as the internal control. Three independent biological repeats showed similar patterns. The band intensities in the top panel were quantified by ImageJ, normalized by the levels of tubulin (bottom panel). The results represent the mean ± SD of three biological replicates. *, $P < 0.05$. (B) Detection of ROS production in the roots of 9-day old seedlings on the ½ MS plate and ½ MS plate with different ROS inhibitors (0.5 mM Ascorbate sodium and 100 nM Diphenyleneiodonium (DPI)) under 1 h submergence. Root samples were stained with 2′,7′-dichlorofluorescein diacetate and visualized with a confocal microscope. The results represent the mean of fluorescence intensity ± SD. **, $P < 0.01$. (C) Western blot showing that phosphorylation of eIF2α was induced in the whole seedlings of Col-0 on ½ MS plates with and without ROS inhibitors under submergence (light). Tubulin was used as the internal control. Three independent biological repeats showed similar patterns.

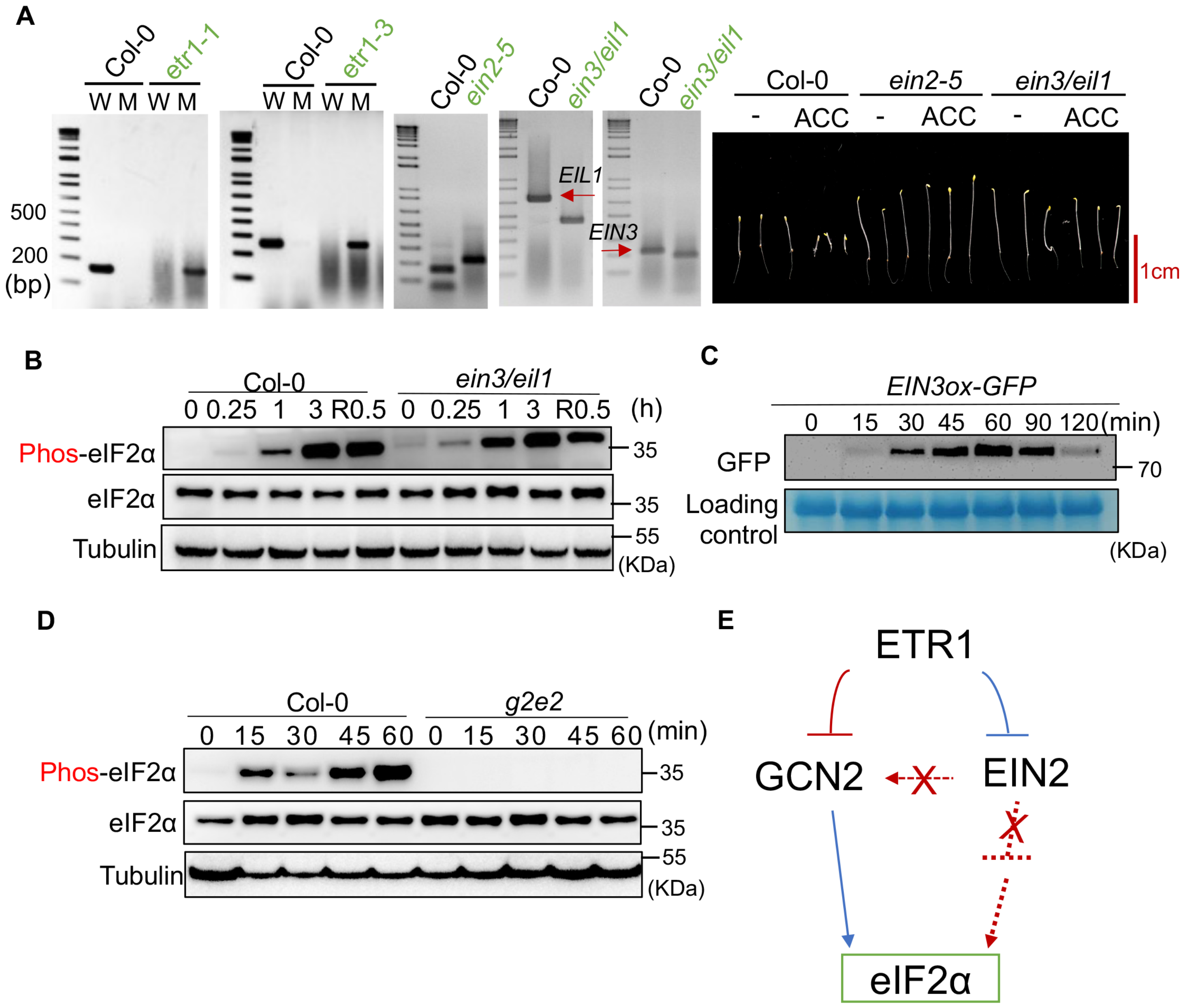

Figure S3. Canonical ethylene signaling was activated under submergence (light). (A) Genotyping of ethylene mutants and triple response tests of ethylene mutants. (Left) Genomic DNA of Col-0, *etr1-1*, *etr1-3*, *ein2-5*, and *ein3/eil1* were amplified to examine the mutant sites. The primers are listed in Supplementary Table S2. W: wild-type allele. M: mutated allele. Red arrow indicates the PCR production of wild type allele. (Right) The triple response tests of *ein2-5* and *ein3/eil1* on the 1/2MS plate with or without ACC under dark. (B) Western blot shows that phosphorylation of eIF2α was induced in the whole seedlings of Col-0 and *ein3/eil1* under submergence (light). Tubulin was used as an internal control. Four independent biological repeats showed similar patterns. (C) Western blot showing the accumulation of EIN3-GFP under submergence (light). The stain of total protein loading was used as the internal control. Two independent biological repeats showed similar patterns. (D) Western blot showing that the phosphorylation of eIF2α was induced in the whole seedling of Col-0 but not in *g2e2* under submergence (light). Tubulin was used as the internal control. Three independent biological repeats show similar patterns. (E) Schematic diagram of ETR1 mediated molecular network, which represses translation under submergence (light). Arrows represent activation; bar-headed lines represent repression; dash lines represent predicted regulation; crosses indicate the experimental results.

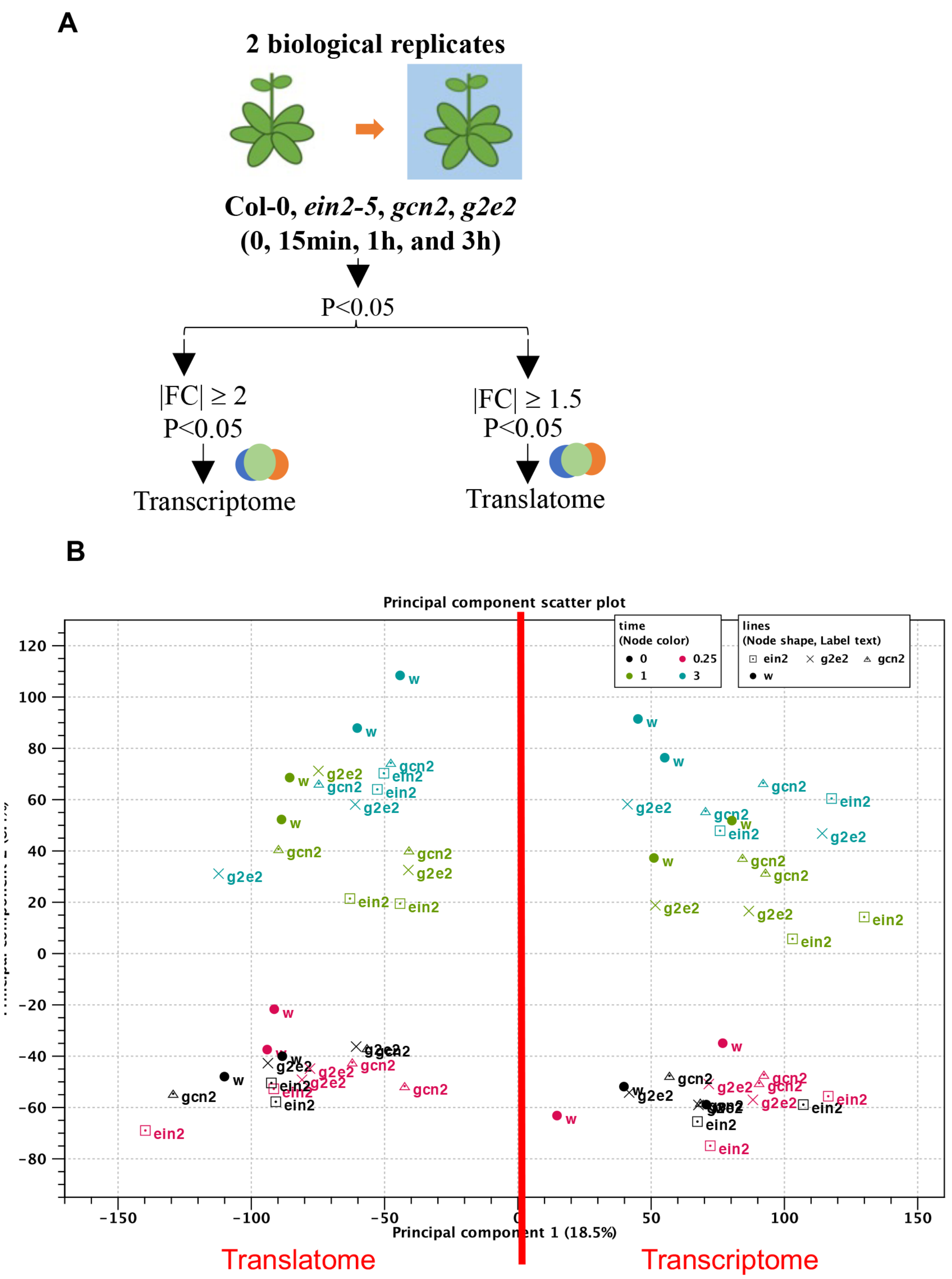

Figure S4. Flowchart of next-generation sequencing analysis. (A) Schematic of experimental procedures and analysis. Two biological replicates of four lines treated with the indicated durations of submergence, were collected to generate transcriptome and translatome profiles. The details of the analysis process are provided in the Methods. (B) The principal component scatter plot shows the separation of the translatome and transcriptome data in PC1 and PC2 dimensions. Different time points and lines are indicated by different colors and shapes.

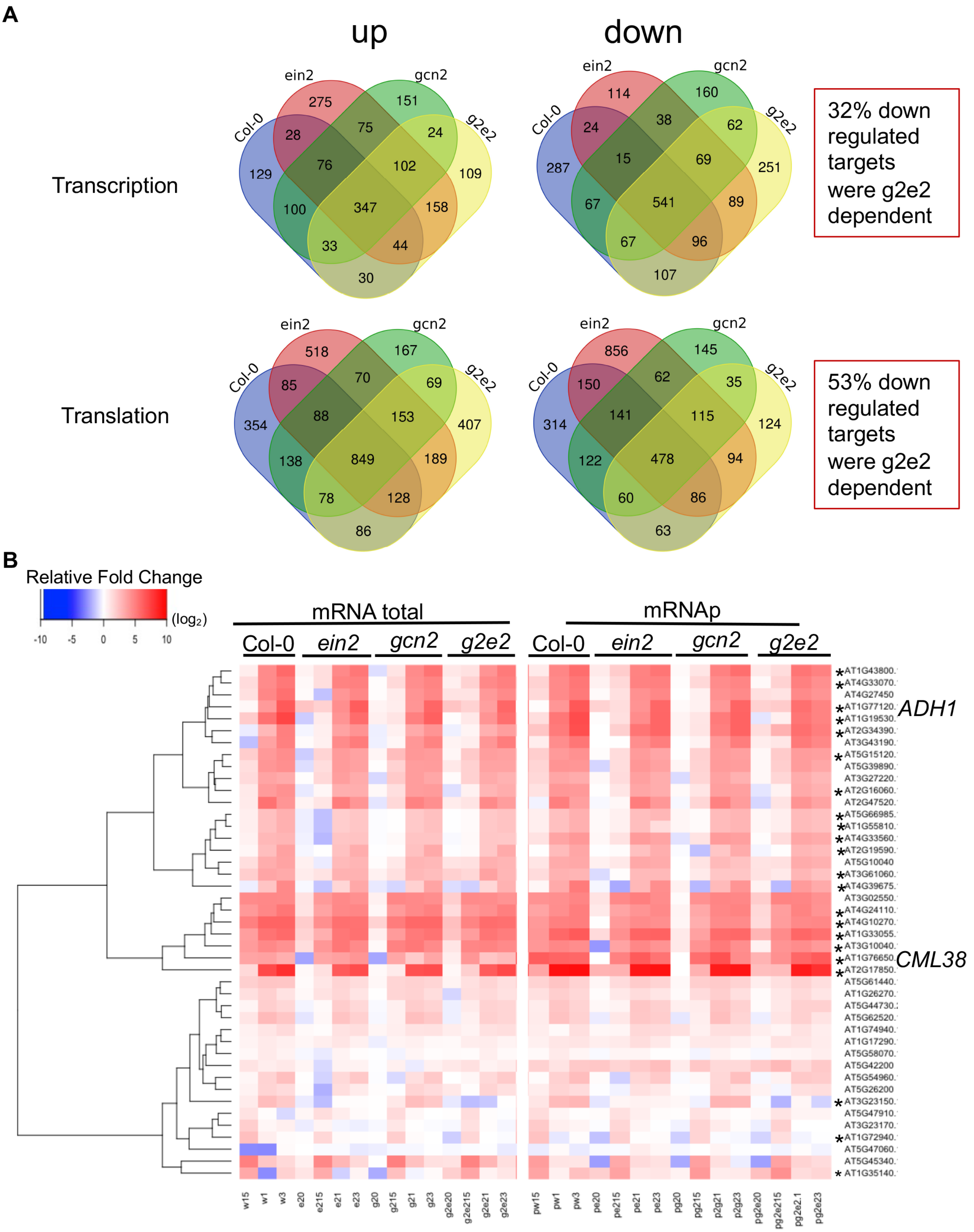

Figure S5. Comparison of the transcriptomes and translatomes of Col-0, *gcn2*, and *ein2*. (A) Venn diagrams showing the overlap between the transcriptomes (upper panel) and translatomes (lower panel) of four lines. The numbers of significantly differentially expressed genes in each line are shown. (B) Heat map showing the transcriptome (left panel) and translatome (right panel) profiles of core hypoxia genes identified in four lines under submergence (41 translationally up regulated genes and 2 transcriptionally repressed genes). *, indicates that translation was disrupted in *g2e2* under submergence.

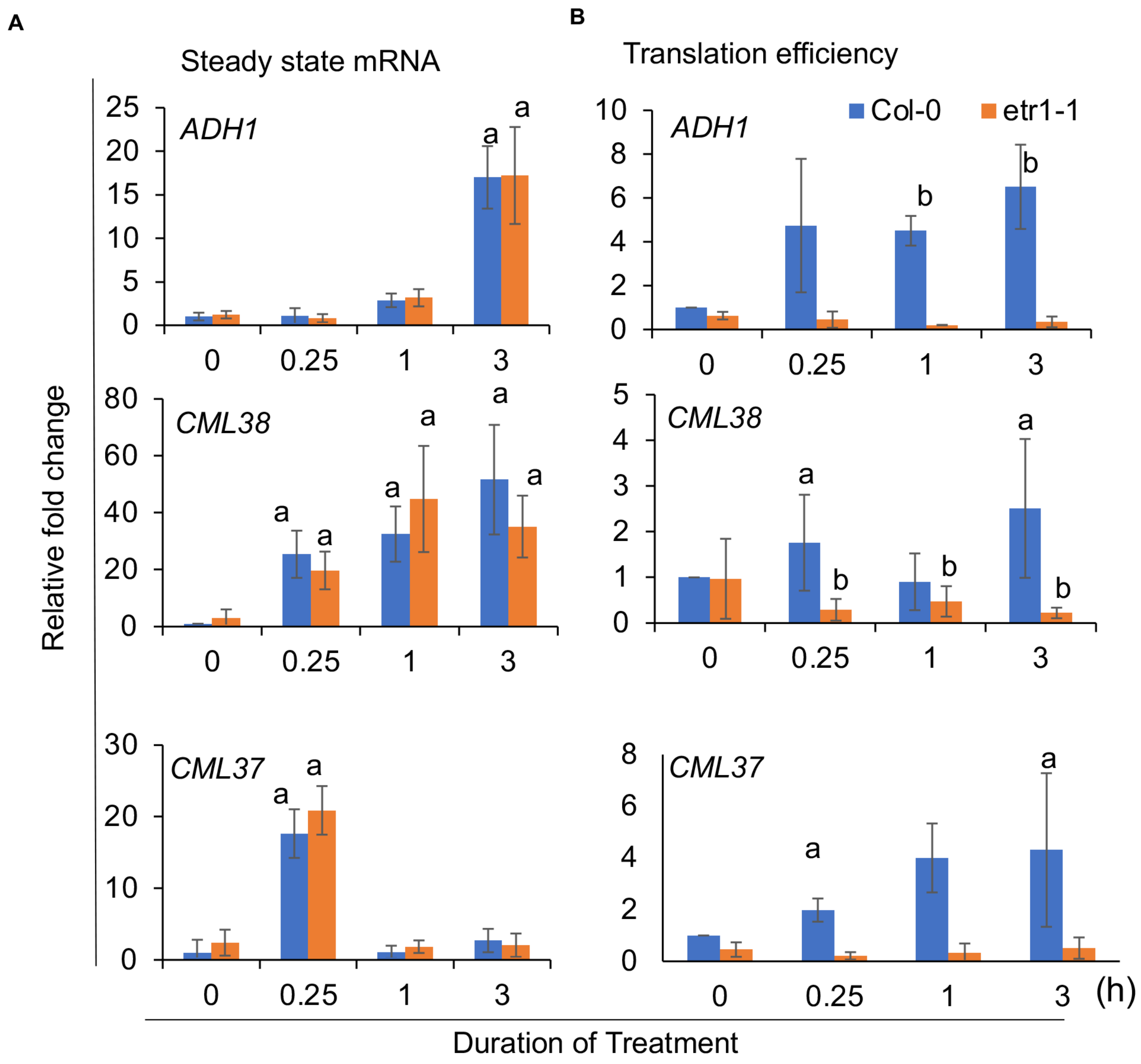

Figure S6. ETR1 is involved in the translation of specific mRNAs under submergence. (A) The transcript levels of hypoxia response genes in the whole seedlings of Col-0 and *etr1-1* were quantified by qPCR, and tubulin was used as an internal control. The relative fold change of each line was normalized with Col-0 under control (time zero). Values represent mean of relative fold change ± SD (4 biological replicates). Different letters represent a significant difference determined by Student's t-test. (B) The translational efficiency of hypoxia response genes in in the whole seedlings of Col-0 and *etr1-1* was quantified by qPCR, and the expression level of mRNA at each indicated time point was used as an internal control. The relative fold change of each line was normalized with Col-0 under control (time zero). Values represent mean of relative fold change ± SD (4 biological replicates). Different letters represent a significant difference determined by Tukey's test.

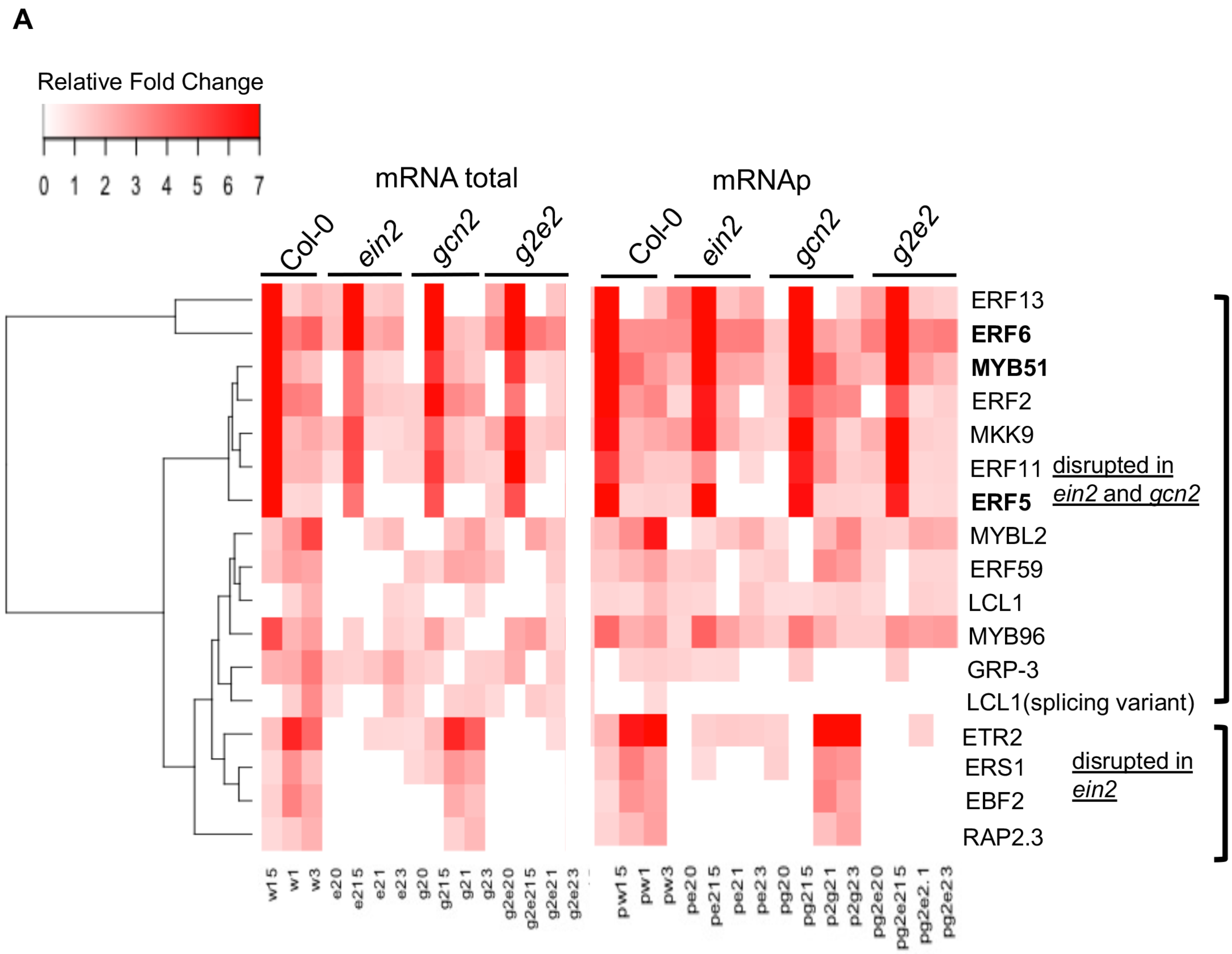

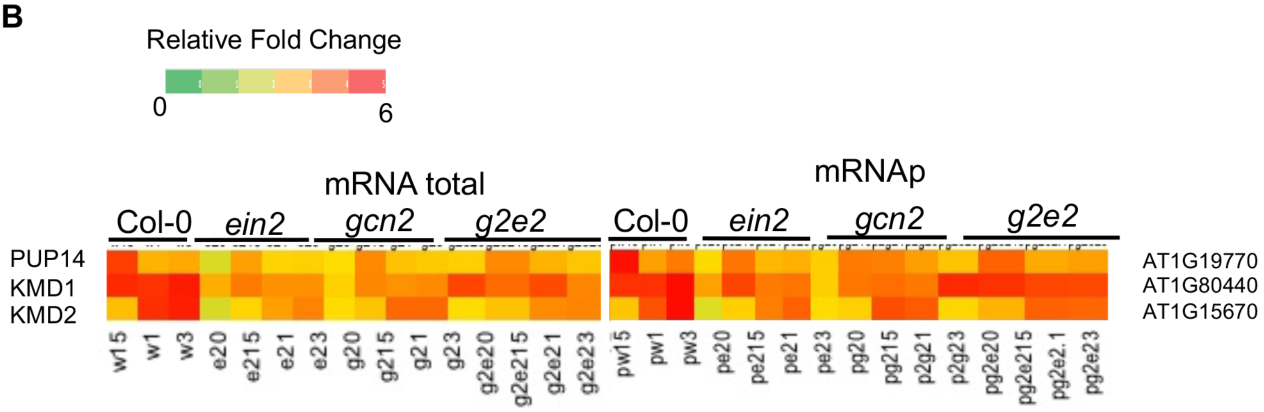

Figure S7. Comparison of ethylene and cytokinin gene expression in *Col-0*, *gcn2*, *ein2*, and *g2e2*. The heat map shows the $mRNA_{total}$ (left panel) and $mRNA_p$ (right panel) levels of (A) ethylene signaling genes and (B) negative regulators of cytokinin signaling in four lines under submergence. The relative fold change in each line at each time point was compared with Col-0 time zero.

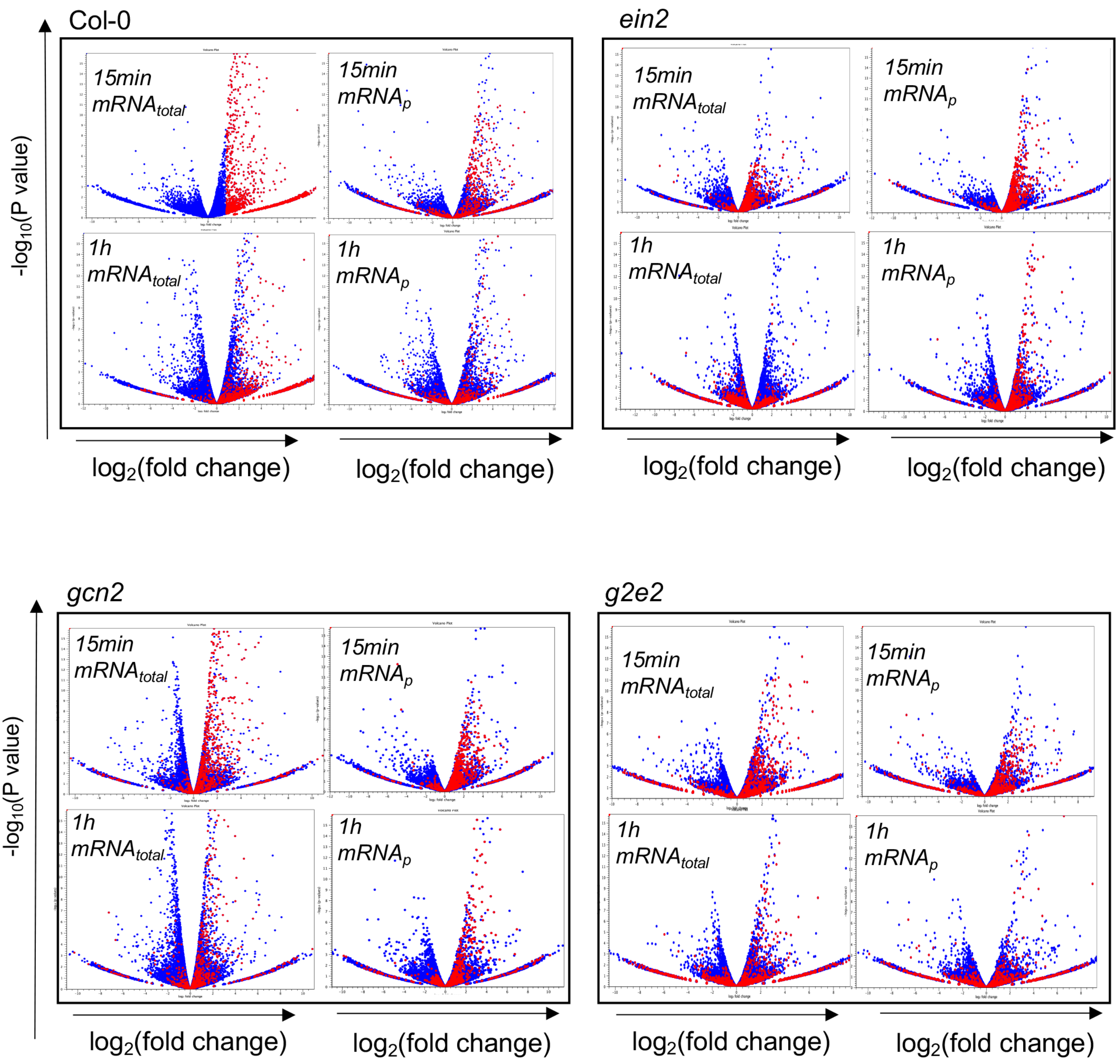

Figure S8. Scatter plots (volcano plots) showing fold changes in $mRNA_{total}$ or $mRNA_p$ level versus P value for four lines under 15 min and 1 h of submergence. Blue dots in each line plot represent individual genes. Red dots indicate genes showing significant upregulation in Col-0.

A

GO biological process complete	Col-0	EIN2	GCN2	GCN2/ EIN2
cellular response to sulfur starvation	34			
glucosinolate biosynthetic process	26	48		
**anthocyanin-containing compound biosynthetic process**	19	> 100	> 100	88
cellular response to blue light	12			
toxin catabolic process	12			
response to insect	11	47		5
response to karrikin	9			
glutathione metabolic process	9			
phenylpropanoid biosynthetic process	7			
response to UV	5			
circadian rhythm	5			
response to red or far red light	4			
regulation of hormone levels	4			
multicellular organism development	2			
response to water deprivation	3			
response to cold	4			8
protein folding	4			12
response to heat	4			
response to jasmonic acid	4			

B

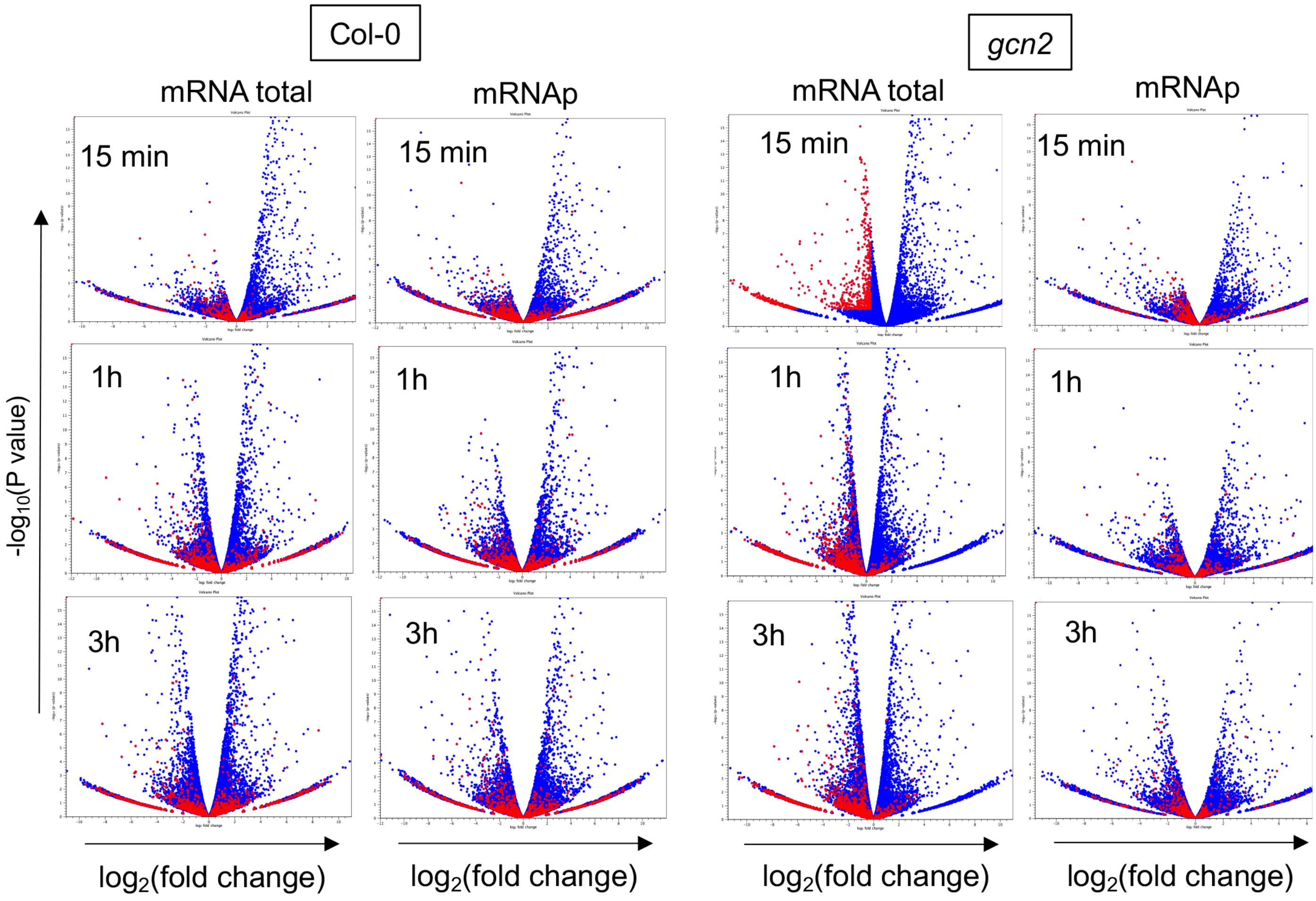

Figure S9. Transcriptome Comparison in 4 lines under submergence. (A) Enriched GO terms (P <0.05, fold enrichment >2) of genes downregulated under submergence in Col-0 but with significantly higher in either *gcn2*, *ein2,* and *g2e2* . The number indicated the enriched fold of each GO. (B) Scatter plots (volcano plots) showing fold changes in $mRNA_{total}$ or $mRNA_p$ level versus P-value in Col-0 and *gcn2* under 15 min, 1 h and 3 h of submergence. Blue dots in each line plot represent individual genes in each line. Red dots indicate genes showing significant downregulation in *gcn2*.

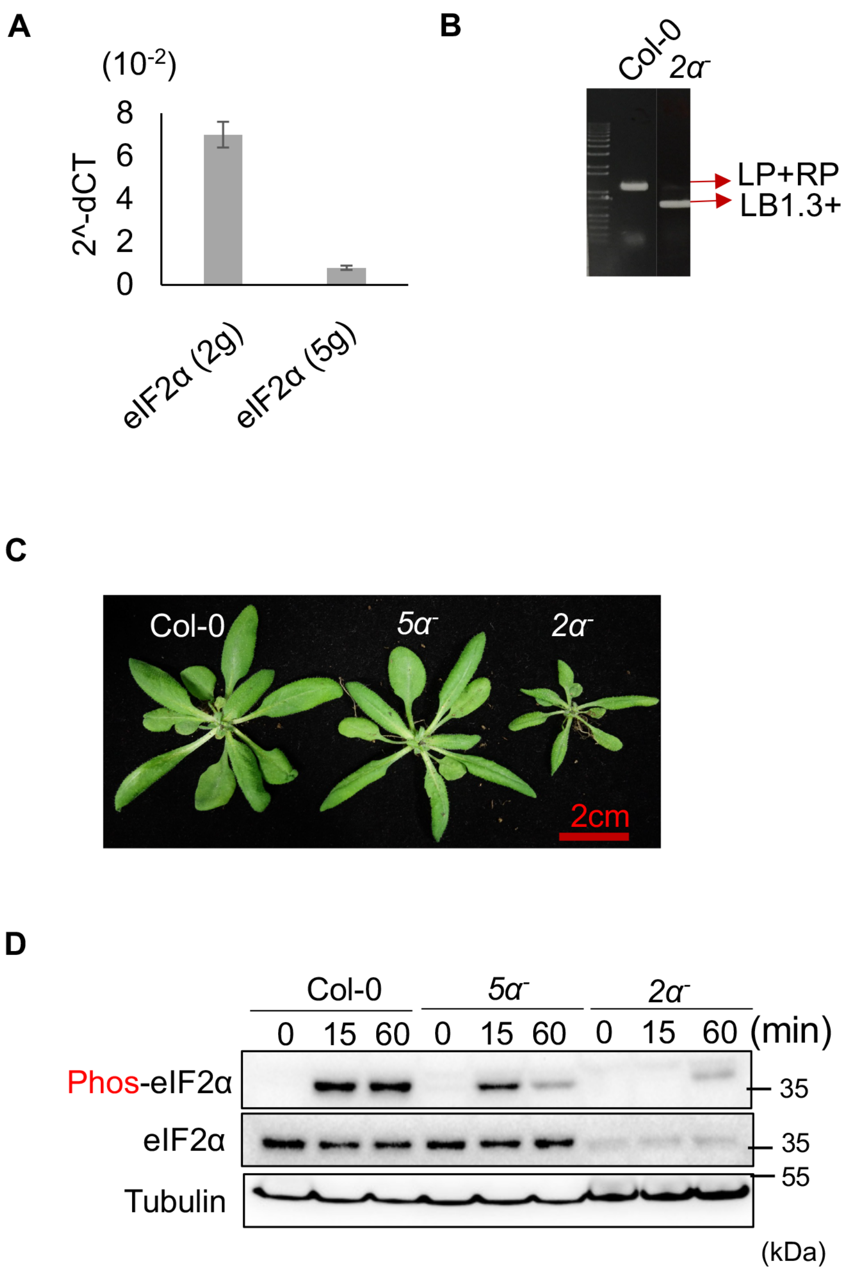

Figure S10. Isolation of the *Arabidopsis eIF2α* knockout lines. (A) The transcript levels of *eIF2α* (*AT2G40290 (eIF2α(2g))* and *AT5G05470 (eIF2α(5g))*) in the whole seedlings of Col-0 were quantified by qPCR, and tubulin was used as the internal control. The data represent the mean ± SD of three biological repeats. (B) Genotyping of the eIF2α mutants, *2α-* and *5α-*. Genomic DNA of Col-0, *2α-* (left panel), and *5α-* (right panel) was amplified with primers LB1.3 and RP to genotype for the T-DNA insertion and the RP and LP primers to genotype for the absence of a T-DNA insertion allele. (C) The phenotypes of 4-week-old Col-0, *2α-*, and *5α-* plants under long-day growth conditions. (D) Western blot demonstrating the phosphorylation and total protein abundance of eIF2α in Col-0 and two *eif2α* lines under submergence (light). Tubulin was used as the internal control. Two independent biological repeats showed similar patterns.

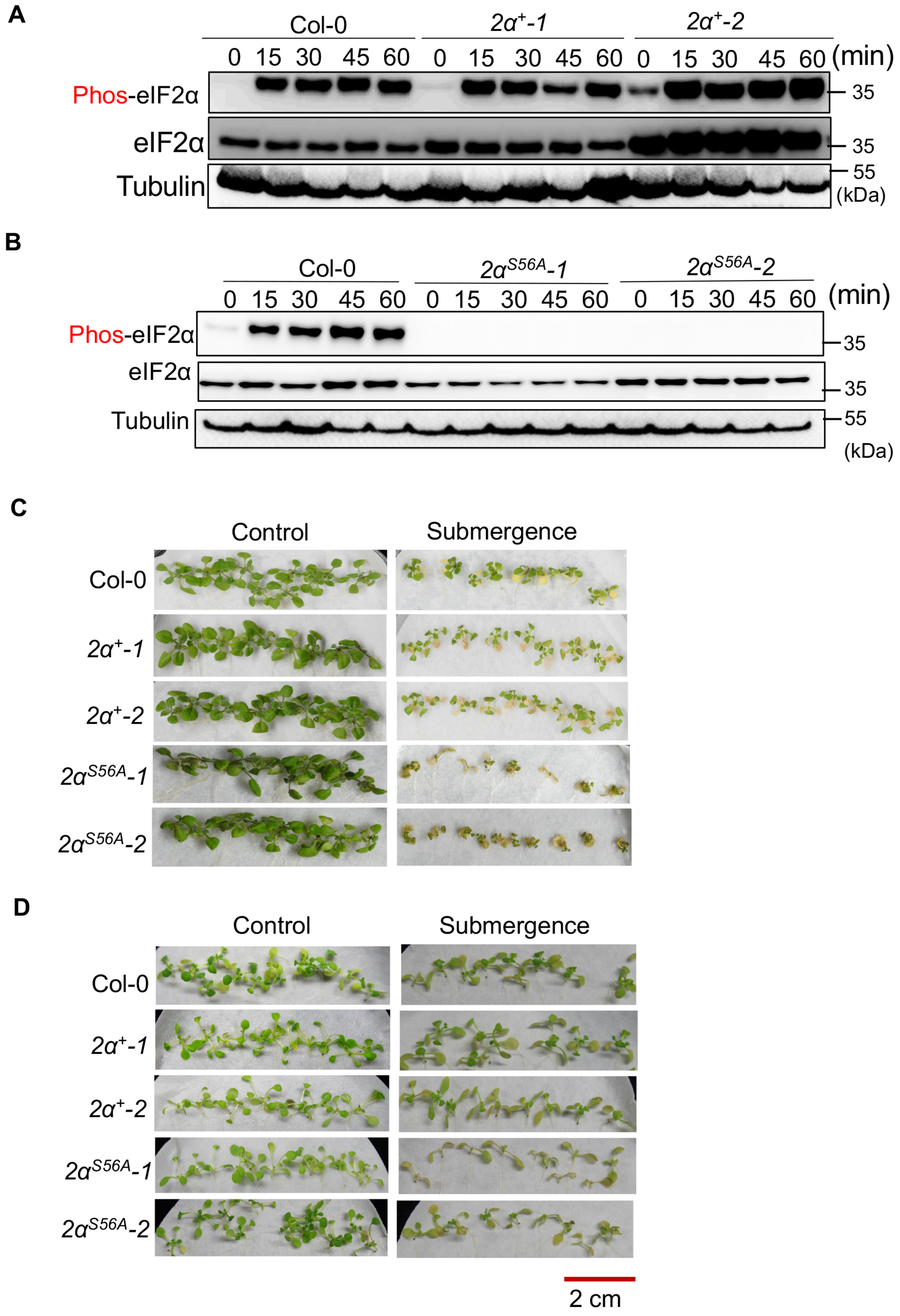

Figure S11. Isolation of the wild-type (*$2\alpha^{+}$*) and S/A (*$2\alpha^{S56A}$*) *eIF2α* lines in *Arabidopsis*. (A-B) Western blot showing the phosphorylation and total protein abundance of eIF2α in the whole seedlings of Col-0 and four *eIF2α* transgenic lines. Tubulin was used as the internal control. Two independent biological repeats showed similar patterns. (C-D) The quantification of green leaf number of 9-day-old Col-0 and *eIF2α* transgenic lines on the third day after (C) 6 days of submergence in the light and (D) 2 days of submergence in darkness.

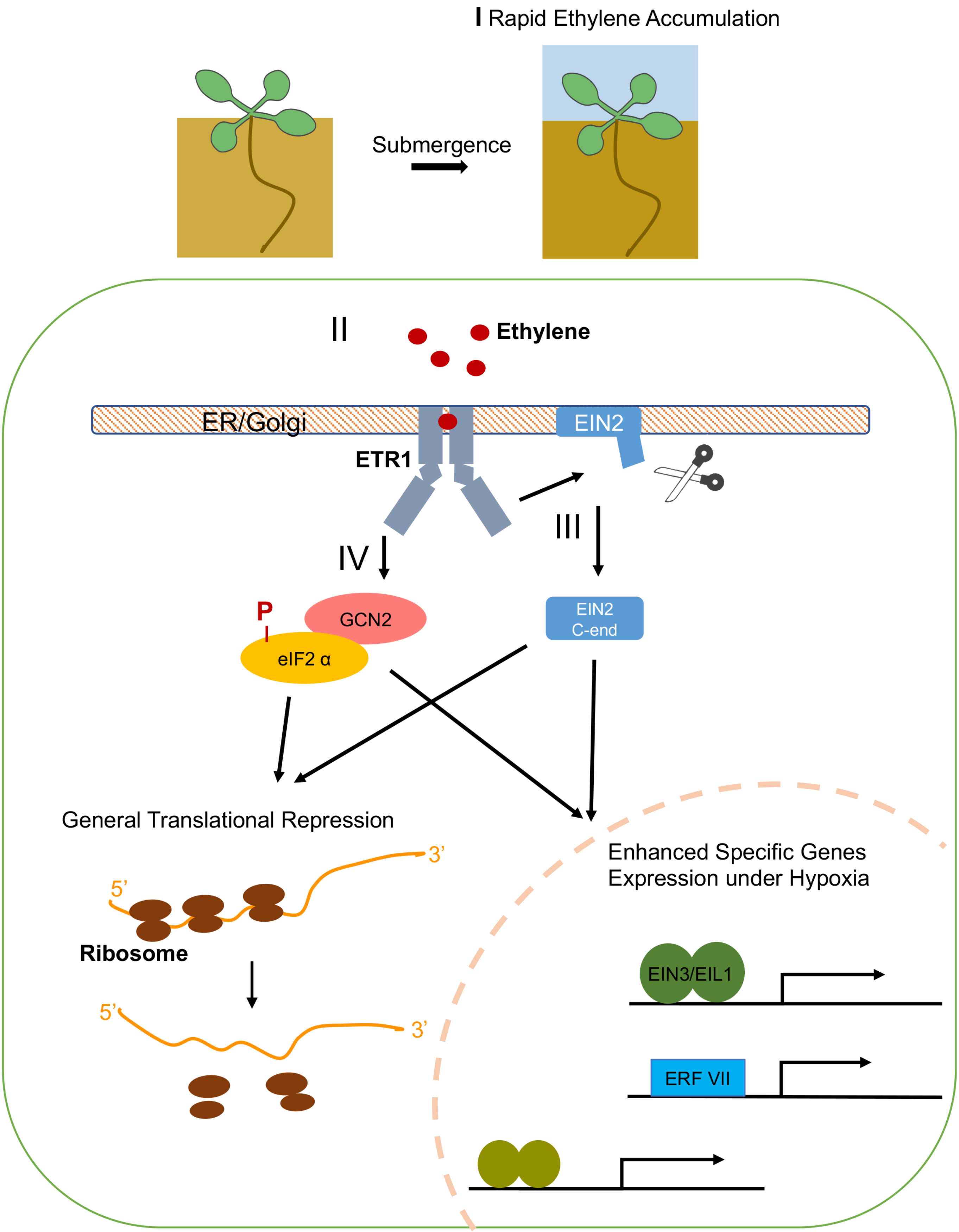

Figure S12. Proposed signaling cascade of ethylene-induced submergence tolerance under submergence. I Under submergence, entrapped ethylene accumulates within minutes in plant tissues. **II** The entrapped ethylene is sensed by ETR1, which leads to activating GCN2 and EIN2 pathways. **III+IV** GCN2 and EIN2 synergistically repress the general translation and prepare the production of specific proteins for the hypoxia upon submergence. GCN2 is activated to phosphorylate eIF2α to reduce the general translation and adjust specific genes expression; The c-end of EIN2 is released to repress the general translation in the cytosol and simultaneously shuttled into the nuclear to trigger either EIN3/EIL1 or ERF VII dependent transcriptional regulations.
